# SRC-YOLOv8n: a lightweight framework for fine-grained apple leaf disease detection with spatial detail preservation and multi-scale feature enhancement

**DOI:** 10.3389/fpls.2025.1709939

**Published:** 2026-02-16

**Authors:** Hanzhi Cui, Chuanlei Song, Conghan Zhong, Peiliang Du, Lihua Xie, Yang Song, Ranran Li, Xiaoliu Jing, Qiuxue Ouyang

**Affiliations:** 1 College of Computer Engineering, Qingdao City University, Qingdao, China; 2 School of Management, Qingdao City University, Qingdao, China; 3 School of Mechanical and Electrical Engineering, Qingdao City University, Qingdao, China

**Keywords:** apple leaf disease detection, lightweight neural network, multi-scale feature fusion, cross-level attention, plant pathology

## Abstract

Apple leaf disease detection is crucial for maintaining crop health and ensuring food security, yet current detection methods face significant challenges in balancing accuracy with computational efficiency. Existing lightweight detection models struggle with spatial detail preservation and multi-scale feature representation when processing complex disease symptoms with subtle visual characteristics. This study presents SRC-YOLOv8n, a lightweight framework that integrates spatial detail preservation and multi-scale feature enhancement for fine-grained apple leaf disease detection. The framework incorporates four key innovations: the Spatial Detail Attention C2f (SDA-C2f) module that preserves critical spatial information through Space-to-Depth Convolution and SpatialGroupEnhance mechanisms, the Reparameterized Generalized Feature Pyramid Network (RepGFPN) that optimizes multi-scale feature fusion through training-inference decoupling, the Cross-Level Local Attention Head (CLLAHead) that enables effective cross-scale feature interaction, and the Inner-IoU loss function that improves bounding box regression accuracy. Comprehensive evaluation on the Plant-Pathology-2021-FGVC8 and AppleLeaf9 datasets demonstrates that SRC-YOLOv8n achieves superior performance with 94.1% precision, 92.3% recall, 96.1% mAP50, and 93.2% F1 score while reducing parameters by 16.6%, computational cost by 19.8%, and model size by 17.7% compared to baseline YOLOv8n. The framework provides an effective solution for real-world agricultural monitoring applications requiring both high accuracy and computational efficiency.

## 1 Introduction

**Figure 1:**
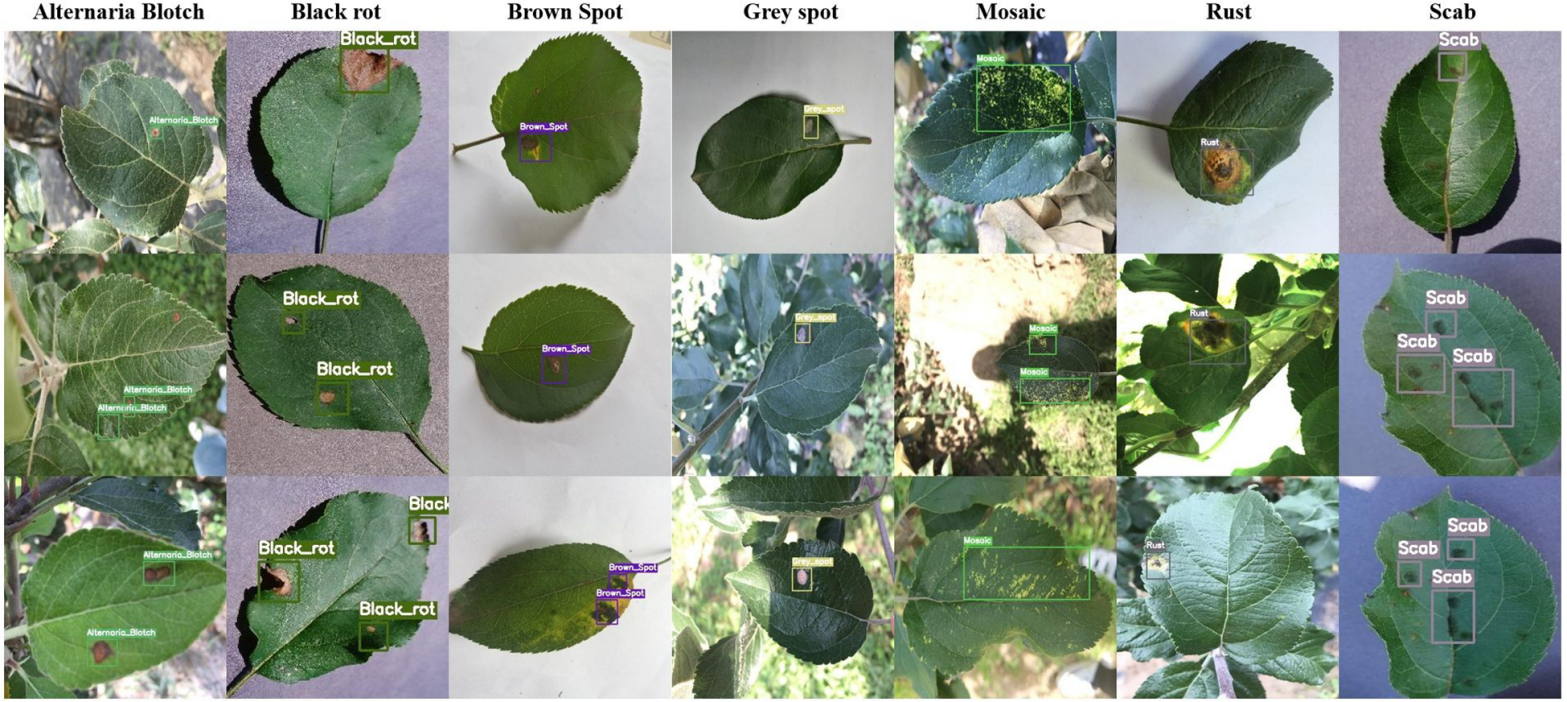
Sample display of seven apple leaf disease types from Plant-Pathology-2021-FGVC8 and AppleLeaf9 datasets: Alternaria Blotch, Black Rot, Brown Spot, Grey Spot, Mosaic, Rust and Scab.

Apple, as one of the most economically valuable fruit crops worldwide, significantly impact agricultural economy and global food supply chains, with their production involving the livelihoods of millions of farmers and food security globally (Dolatabadian et al., 2025; Upadhyay et al., 2025). However, as shown in **Figure 1**, apple leaf diseases such as alternaria blotch, black rot, brown spot, grey spot, mosaic, rust, and scab pose severe threats to tree health, directly compromising photosynthetic capacity and leading to reduced yields and deteriorated fruit quality (Liu and Li, 2024; Li et al., 2024b). Even with regular pesticide applications, apple leaf diseases persist, making accurate and timely identification crucial for effective disease control, maintaining apple quality and production levels, and ultimately achieving economic and environmental benefits.

Traditional monitoring methods relying on manual field inspections are insufficient for modern intensive orchard management. Current apple leaf disease detection primarily depends on field observations and experiential judgments by agricultural experts, which suffers from inherent limitations including high labor intensity, time consumption, and susceptibility to personnel expertise levels, visual fatigue, and subjective assessments (Xu and Wang, 2023; Alhwaiti et al., 2025). During early disease stages, apple leaves are typically small and densely distributed, severely limiting human visual sensitivity and often missing optimal treatment timing. Furthermore, manual detection in large-scale orchards has limited coverage, creating monitoring blind spots, resulting in inefficient processes constrained by expert knowledge and experience, prone to misdiagnosis and omissions, unable to meet real-time monitoring demands.

Apple leaf diseases present diverse symptoms that are easily confused in early stages. While deep learning has advanced plant disease detection, existing lightweight models face critical trade-offs: spatial information loss during downsampling, insufficient multi-scale feature fusion, and suboptimal cross-scale information interaction, particularly affecting small lesion and medium-scale disease feature detection (Shoaib et al., 2025).

The objective of this study is to develop a lightweight yet robust framework for fine-grained apple leaf disease detection by addressing the fundamental challenges in spatial detail preservation, multi-scale feature fusion, and cross-level information interaction. We utilized two publicly available datasets: Plant-Pathology-2021-FGVC8 (Thapa et al., 2021) and AppleLeaf9 (Yang et al., 2022), from which we selected and annotated seven major apple leaf disease types including alternaria blotch, black rot, brown spot, grey spot, mosaic, rust, and scab to construct a comprehensive evaluation dataset. To overcome the limitations of traditional YOLOv8n in handling subtle disease features and complex multi-scale lesion distributions, we propose SRC-YOLOv8n, which integrates four key innovations specifically designed to address the aforementioned challenges: (1) SDA-C2f (Spatial Detail Attention C2f) module for lossless spatial detail preservation through Space-to-Depth Convolution and spatial group enhancement mechanisms, effectively addressing the spatial information loss problem in conventional downsampling operations; (2) RepGFPN (Reparameterized Generalized Feature Pyramid Network) for efficient multi-scale feature fusion through training-inference decoupling reparameterization strategy, enabling comprehensive feature integration across different scales while maintaining computational efficiency; (3) CLLAHead (Cross-Level Local Attention Head) for enhanced cross-level attention mechanisms, introducing local attention windows and irregularity calculations to improve medium-scale disease feature representation; and (4) Inner-IoU (Inner Intersection over Union) loss for improved bounding box regression accuracy through adaptive auxiliary bounding box scaling, particularly beneficial for irregular disease regions. Unlike existing methods that focus on single-aspect improvements, our framework provides a holistic solution by synergistically integrating spatial preservation, multi-scale fusion, and attention mechanisms, making it particularly suitable for resource-constrained agricultural monitoring systems requiring real-time processing capabilities. The proposed SRC-YOLOv8n aims to achieve superior detection performance while maintaining computational efficiency, thereby providing a practical and effective solution for intelligent agricultural disease monitoring systems and contributing to enhanced productivity and economic sustainability in apple cultivation.

## 2 Related work

The emergence of computer vision technology has provided new pathways for agricultural disease detection. Early research employed traditional machine learning methods combined with hand-crafted feature extraction for plant disease identification, achieving certain results in simple backgrounds but lacking robustness in complex natural environments (Aghajanpoor et al., 2023). The rapid development of deep learning technology, particularly convolutional neural network-based methods, has shown tremendous potential in plant disease detection, bringing revolutionary breakthroughs to plant phenotyping and disease detection (Sujatha et al., 2025; Zhu et al., 2024). Compared to traditional methods, deep learning demonstrates significant advantages in plant phenotyping: automatic learning and extraction of complex visual features without requiring manual feature descriptor design; powerful nonlinear mapping capabilities for handling target recognition tasks in complex backgrounds; and significantly improved generalization and robustness through large-scale data training.

### 2.1 Deep learning-based plant disease detection

Object detection algorithms have provided strong technical support for agricultural disease detection. The YOLO series models, with their end-to-end detection architecture and real-time processing capabilities, have gained widespread attention in agricultural applications. Recent research has explored deep learning solutions specifically for disease detection. Recent studies (Ullah et al., 2024; Bonkra et al., 2024) have explored YOLO-based solutions for disease detection, improving accuracy through attention mechanisms and multi-scale feature fusion.

YOLOv8 has achieved significant improvements in detection accuracy, inference speed, and model lightweightness. Compared to YOLOv5, YOLOv8 adopts more efficient backbone networks and neck structures, introducing improved loss functions and training strategies, performing particularly well in small target detection tasks. Tarasiuk et al. demonstrated that YOLOv8 achieved improved mAP values compared to YOLOv7 in plant disease detection tasks while maintaining faster inference speeds (Tarasiuk et al., 2025). The latest YOLOv10 and YOLOv11 models have introduced end-to-end detection mechanisms and further architectural optimizations, providing new baselines for agricultural applications (Wang et al., 2024). Selecting YOLOv8 as the base architecture allows full utilization of its advanced network design while enabling targeted improvements to adapt to the specific requirements of apple leaf disease detection.

### 2.2 Lightweight detection models for agricultural applications

The balance between model lightweightness and detection accuracy remains a critical issue in current research, particularly for mobile deployment and edge computing scenarios (Shoaib et al., 2025). Recent studies have made significant progress in developing lightweight detection frameworks for plant disease identification. Liu and Li introduced asymmetric ShuffleBlocks and BSConv, achieving 91.08% mAP on the MSALDD dataset at 122 FPS (Liu and Li, 2024). Xu and Wang compressed the model size by replacing common convolution with group convolution in the SPPCSPC module, achieving 90.2% accuracy with only 6.1 GFLOPs (Xu and Wang, 2023). Gao et al. proposed a lightweight YOLOv8 model incorporating GhostConv and C3Ghost modules, reducing parameters while maintaining detection accuracy (Gao et al., 2024). Li et al. developed YOLO-Leaf utilizing Dynamic Snake Convolution (DSConv) and BiFormer attention mechanisms, achieving mAP50 scores of 93.88% and 95.69% on FGVC7 and FGVC8 datasets, respectively (Li et al., 2024b).

More recently, Wang et al. introduced ELM-YOLOv8n with restructured neck networks and lightweight detection heads, achieving 96.7% mAP50 with improved performance on small target diseases (Wang et al., 2025). Zhang et al. proposed LightYOLO-AppleLeafDx incorporating Slim-Neck, SPD-Conv, and SAHead modules, achieving 96.5% mAP50 with only 5.2 MB model size and 107.2 FPS detection speed. These lightweight approaches demonstrate the feasibility of deploying efficient detection models on resource-constrained devices while maintaining high accuracy, which aligns with the objectives of the present study.

### 2.3 Multi-scale feature fusion strategies

Recent advances in reparameterization techniques have further improved feature fusion efficiency. Chu et al. introduced RepVGG-style reparameterization for GFPN, enabling rich multi-branch architectures during training while maintaining single-path efficiency during inference (Chu et al., 2024). Li et al. developed Adaptive Reparameterized GFPN for small target detection, demonstrating superior performance in water surface object detection tasks. Zhang et al. proposed PARE-YOLO with restructured neck networks incorporating multi-scale attention mechanisms, achieving 5.9% improvement in mAP on VisDrone2019 dataset (Zhang et al., 2025). These reparameterization-based approaches provide valuable insights for designing efficient multi-scale feature fusion modules in agricultural disease detection.

### 2.4 Attention mechanisms for enhanced detection

Attention mechanisms have proven essential for improving detection performance by selectively focusing on relevant features. Zhang et al. proposed YOLO-ACT incorporating adaptive cross-layer integration methods, demonstrating improved performance in apple leaf disease detection through enhanced attention mechanisms (Zhang et al., 2024). Wu et al. introduced multi-scale feature fusion with attention mechanisms for crowded object detection, effectively identifying key regions in dense scenes (Wu et al., 2024). Lee et al. developed cross-guided attention for multi-modal object detection, achieving state-of-the-art performance through complementary interactions between modalities (Lee et al., 2024).

Recent work on cross-level attention has shown particular promise for agricultural applications. Li et al. proposed multi-scale coupled attention for visual object detection, enhancing feature representation through cross-scale feature interaction (Li et al., 2024a). Zhu et al. introduced Transformer Prediction Heads with Convolutional Block Attention Module (CBAM), optimizing cross-scale object discrimination in dense object scenes. Zhang et al. developed efficient attention modules for aerial image small-target detection, significantly improving detection performance in cluttered backgrounds. These attention-based approaches demonstrate the potential of selective feature enhancement for improving disease detection accuracy.

### 2.5 Advanced loss functions for bounding box regression

Bounding box regression loss functions significantly impact detection performance, particularly for objects with irregular shapes and varying sizes. Traditional IoU loss and its variants, including GIoU, DIoU, and CIoU, have established the foundation for IoU-based optimization (Wang and Song, 2021). Zhang et al. proposed Focal and Efficient IoU (EIoU) loss, explicitly measuring discrepancies in overlap area, central point, and side length to accelerate convergence (Zhang et al., 2022). Tong et al. introduced Wise-IoU (WIoU) with dynamic non-monotonic focusing mechanism, evaluating anchor box quality based on outlier degree rather than IoU values (Tong et al., 2023).

Recent advances have further refined IoU-based losses for improved convergence and accuracy. Zhang et al. proposed Inner-IoU loss utilizing auxiliary bounding boxes with adaptive scaling factors, enabling faster convergence for both high and low IoU samples (Zhang et al., 2023). Liu et al. developed Gaussian-IoU loss for PCB component detection, improving bounding box regression accuracy through probabilistic formulation (Liu et al., 2022). Li et al. introduced Generalized Focal Loss with distribution-based bounding box regression, transforming point estimation into distribution estimation for improved localization (Li et al., 2020). These advanced loss functions provide theoretical foundation for designing effective bounding box regression strategies in disease detection tasks.

### 2.6 Research gap and motivation

Despite significant progress in plant disease detection, existing methods face challenges in balancing detection accuracy with computational efficiency for real-world deployment. Current lightweight models often sacrifice spatial detail preservation during aggressive downsampling operations, leading to reduced accuracy for detecting subtle disease symptoms. Multi-scale feature fusion strategies, while effective, frequently suffer from computational redundancy and insufficient cross-scale information interaction. Detection heads in existing frameworks lack effective mechanisms for cross-level feature interaction, resulting in suboptimal performance for medium-scale disease features that are prevalent in apple leaf diseases.

This study addresses these limitations by proposing SRC-YOLOv8n, which integrates spatial detail preservation, efficient multi-scale feature fusion through reparameterization, cross-level attention mechanisms, and advanced bounding box regression strategies. The proposed framework aims to achieve superior detection performance while maintaining computational efficiency, thereby providing a practical solution for intelligent agricultural disease monitoring systems.

The **Table 1** summarizes key characteristics of recent works in lightweight apple leaf disease detection. Unlike existing methods that focus on single-aspect improvements (either backbone optimization, feature fusion enhancement, or attention mechanisms), SRC-YOLOv8n integrates innovations across all components—backbone, neck, detection head, and loss function—achieving superior accuracy-efficiency balance specifically optimized for fine-grained apple disease characteristics.

**Table 1:** Summary of related works in lightweight apple leaf disease detection.

Study	Base model	Key innovation	Advantages	Limitations	mAP50 (%)
Lightweight backbone improvements					
Liu and Li (2024)	YOLOv5	Asymmetric ShuffleBlocks + BSConv	High FPS (122), compact size	Limited spatial detail preservation	91.08
Xu and Wang (2023)	ALAD-YOLO	Group convolution in SPPCSPC	Low GFLOPs (6.1)	Accuracy trade-off (90.2%)	90.2
Gao et al. (2024)	YOLOv8	GhostConv + C3Ghost	Parameter reduction	Insufficient for subtle symptoms	92.3
Wang et al. (2025)	ELM-YOLOv8n	Restructured neck + lightweight head	Small target detection	Complex architecture	96.7
Multi-scale feature fusion methods					
Li et al. (2024b)	YOLO-Leaf	DSConv + BiFormer attention	Multi-scale fusion	Heavy model (12.3M params)	95.69
Zhang et al. (2024)	LightYOLO	Slim-Neck + SPD-Conv + SAHead	Balanced accuracy-efficiency	Limited cross-scale interaction	96.5
Chu et al. (2024)	RepVGG-GFPN	RepVGG reparameterization	Training-inference decoupling	General object detection focus	–
Zhang et al. (2025)	PARE-YOLO	Multi-scale attention in neck	Aerial image optimization	Not optimized for plant diseases	–
Attention-enhanced detection heads					
Zhang et al. (2024)	YOLO-ACT	Adaptive cross-layer integration	Cross-layer feature fusion	Computational overhead-	–
Wu et al. (2024)	Multi-scale YOLO	Multi-scale attention fusion	Crowded object detection	Not for agricultural applications	–
Li et al. (2024a)	MS-YOLO	Multi-scale coupled attention	Visual object detection	General purpose design	–
Advanced loss functions					
Zhang et al. (2023)	Inner-IoU	Auxiliary bounding box scaling	Adaptive convergence	Requires careful tuning	–
Zhang et al. (2022)	EIoU	Explicit distance measurement	Fast convergence	Limited for irregular shapes	–
Tong et al. (2023)	WIoU	Dynamic focusing mechanism	Outlier handling	Complex optimization	–
Ours	SRC-YOLOv8n	SDA-C2f + RepGFPN + CLLAHead + Inner-IoU	Lossless spatial preservation, efficient multi-scale fusion, cross-level attention	Apple disease specific	96.1

## 3 Materials and methods

### 3.1 Experimental design

**Figure 2:**
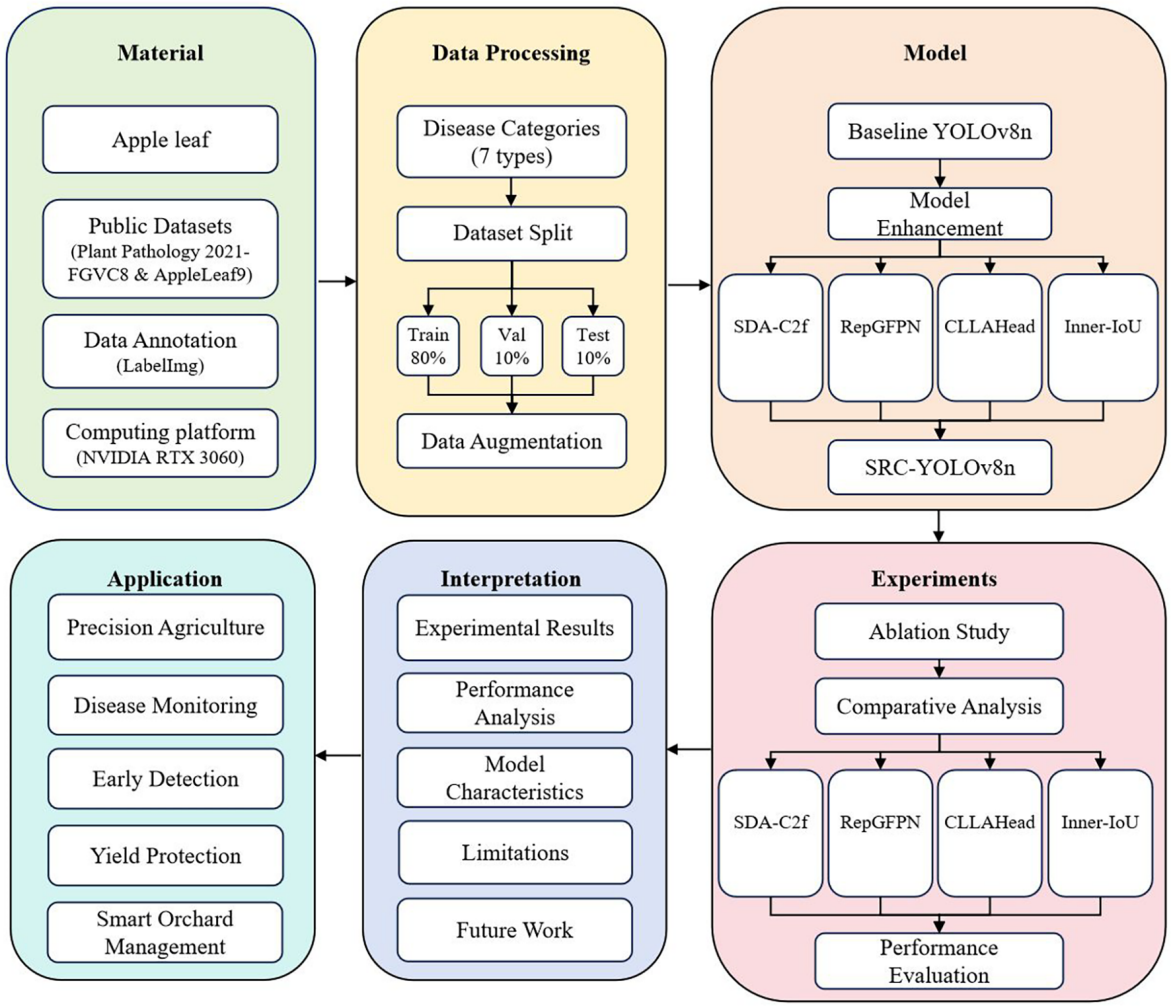
Experimental, technical design, and workflows. The figure illustrates the six main stages: (1) Data Collection Preparation, (2) Dataset Construction Augmentation, (3) Model Architecture Design (SDA-C2f, RepGFPN, CLLAHead, Inner-IoU), (4) Training Optimization, (5) Performance Evaluation Analysis, (6) Practical Application Assessment.

This study is divided into six main parts, as shown in **Figure 2**. Firstly, we collected necessary materials, including apple leaf samples, public datasets (Plant-Pathology-2021-FGVC8 (Thapa et al., 2021) and AppleLeaf9 (Yang et al., 2022)), data annotation tools, and computing platforms (NVIDIA RTX 3060), for tasks such as disease sample collection, data annotation, model training, and performance evaluation. Next, we conducted data processing and classified apple leaf diseases into 7 categories. We divided the training set, validation set, and testing set in a ratio of 8:1:1, and used data augmentation techniques to improve the model’s generalization ability. Subsequently, we enhanced the YOLOv8n baseline model by integrating improved modules such as SDA-C2f, RepGFPN, CLLAHead, and Inner-IoU to accurately identify different types of apple leaf diseases. We have designed a comprehensive experimental plan, including ablation studies and comparative analysis, to evaluate the performance of the improved model compared to other advanced models. We conducted a thorough analysis of the experimental results, including performance analysis, model feature analysis, and limitations discussion, and explored future research directions. Finally, we analyzed the potential application value of the developed model in precision agriculture, disease monitoring, early detection, yield protection, and smart orchard management. The apple leaf disease detection model based on improved YOLOv8n developed in this study provides effective technical support for modern agricultural intelligence.

### 3.2 Dataset construction

The experimental datasets were sourced from the publicly available datasets Plant-Pathology-2021-FGVC8 and AppleLeaf9. Both datasets contain a large number of high-quality images of apple leaf diseases, from which seven leaf diseases were selected for labeling, namely alternaria blotch, black rot, brown spot, grey spot, mosaic, rust, and scab, with more than 90% of the images collected in natural orchard environments. These seven diseases are prevalent on apple leaves, resulting in significant detrimental effects on agricultural productivity, and they are representative in terms of symptom presentation, covering different lesion types such as spotting, rotting, browning, and rusting patterns.

**Figure 3:**
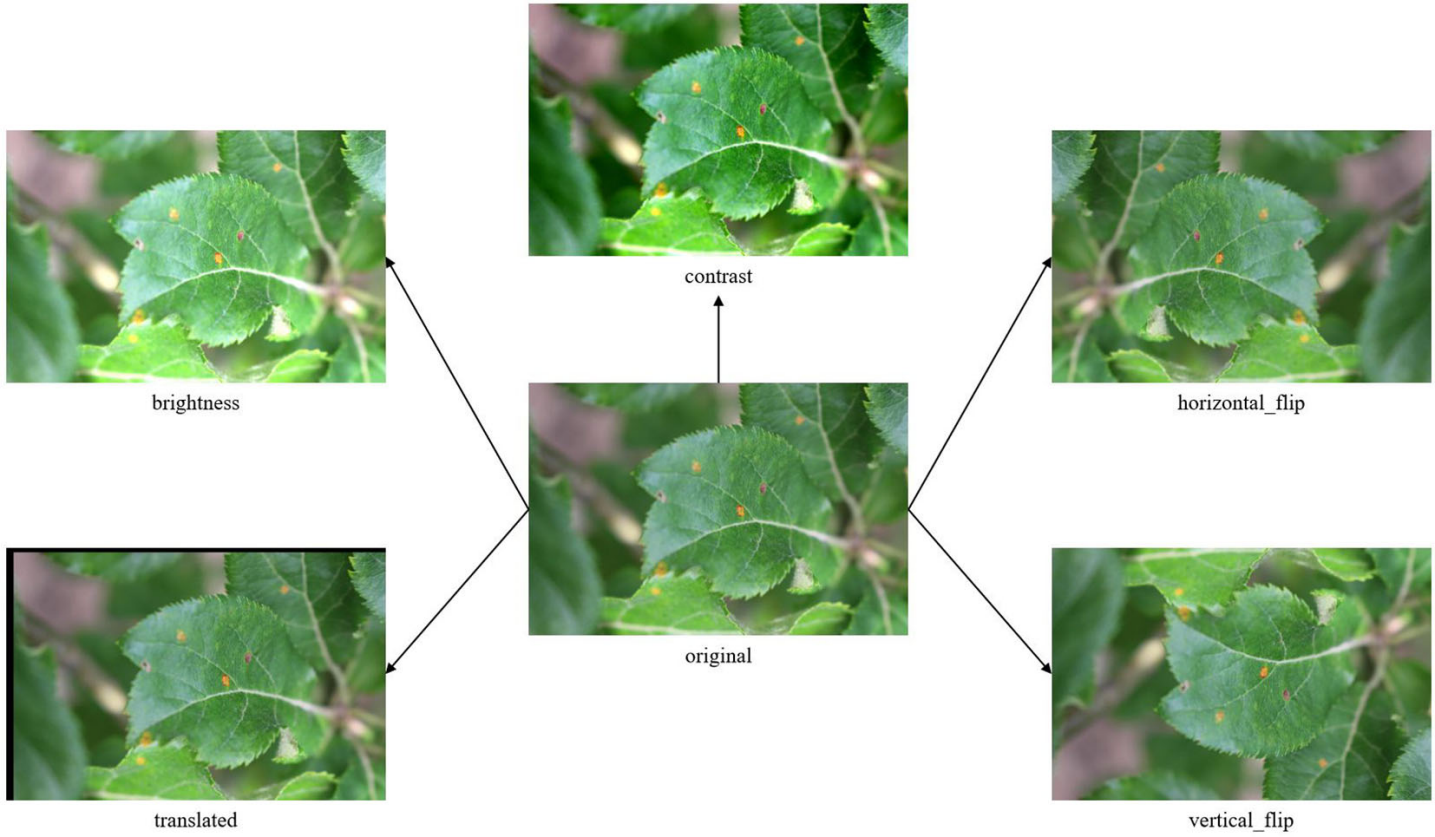
An example of the enhancement methods. The augmentation techniques simulate various real-world conditions: horizontal/vertical flipping represents different leaf orientations, translation accounts for spatial variations, contrast adjustment handles varying lighting conditions, and brightness adjustment addresses different time-of-day scenarios encountered in field applications.

A total of 3,555 images were labeled using the LabelImg annotation tool (Tzutalin, 2015). The bounding box annotation approach was employed to precisely delineate disease regions for all seven categories, with particular attention paid to accurately capturing the boundaries of lesions while maintaining consistency in annotation standards across different disease types. The dataset was augmented through data enhancement techniques. To prevent both the original and enhanced images from appearing simultaneously in the training and validation sets, the original images were initially divided into training, validation, and test sets in a ratio of approximately 8:1:1. Our model requires a substantial amount of data to capture the subtle differences between different diseases, and this ratio ensures that the model has sufficient data for training while maintaining appropriate amounts of data for validation and testing. Subsequently, the images and labels were augmented using five techniques: horizontal flipping, vertical flipping, translation, contrast adjustment, and brightness adjustment. The enhanced dataset obtained is named Apple-Disease-Detection Dataset. An example of the enhancement methods is illustrated in **Figure 3**. The numbers and sources of the images are presented in **Table 2**.

**Table 2:** Distribution of apple leaf disease images in the Apple-Disease-Detection Dataset.

Disease name	Image source	Original	Enhanced train	Enhanced val	Enhanced test	Enhanced total
Alternaria Blotch	2021-fgvc8 & AppleLeaf9	411	1256	155	162	1573
Black rot	2021-fgvc8 & AppleLeaf9	621	760	96	102	958
Brown Spot	2021-fgvc8 & AppleLeaf9	435	551	67	60	678
Grey Spot	2021-fgvc8 & AppleLeaf9	370	1060	123	140	1323
Mosaic	2021-fgvc8 & AppleLeaf9	375	1090	144	140	1374
Rust	2021-fgvc8 & AppleLeaf9	713	896	128	99	1123
Scab	2021-fgvc8 & AppleLeaf9	630	825	91	103	1019
Total		**3,555**	**6,438**	**804**	**806**	**8,048**

Bold values indicate the highest number of images for each disease category in the final enhanced dataset after data augmentation.

### 3.3 SRC-YOLOv8n

**Figure 4:**
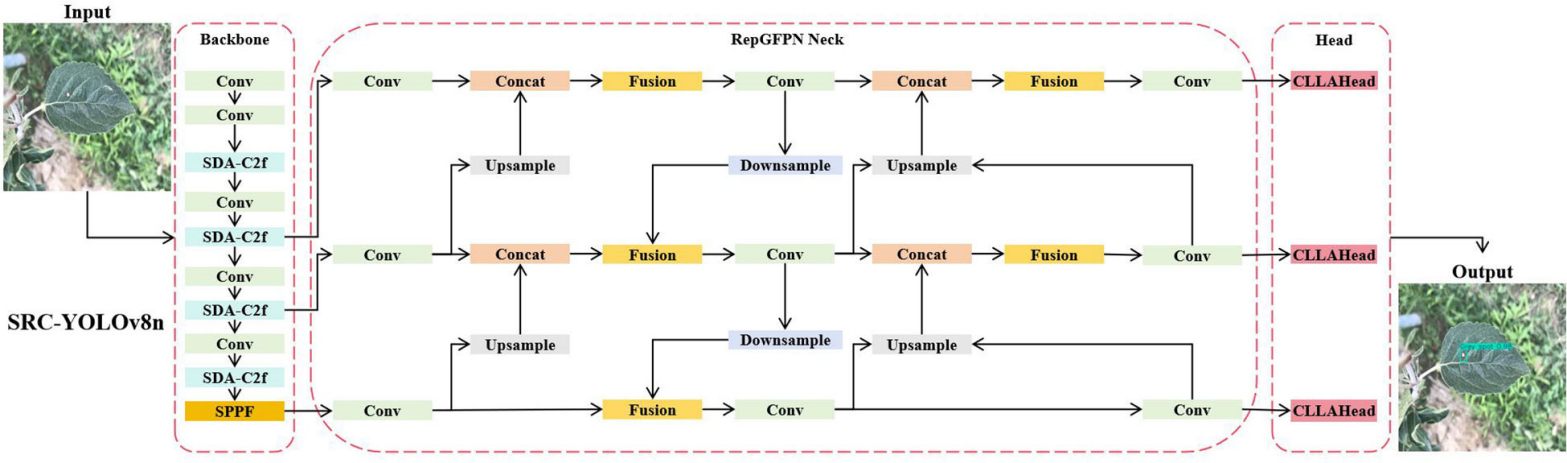
SRC-YOLOv8n network structure. The framework integrates four key innovations: SDAC2f for spatial detail preservation in the backbone, RepGFPN for efficient multi-scale feature fusion in the neck, CLLAHead for cross-level attention enhancement in the detection head, and Inner-IoU loss for improved bounding box regression. The input image (640×640) is processed through multiple stages with varying feature map resolutions.

To address the inherent limitations of YOLOv8n in apple leaf disease detection, particularly the challenges of spatial information loss, inadequate multi-scale feature fusion, and suboptimal bounding box regression for fine-grained disease features, we propose SRC-YOLOv8n (SDA-C2f-RepGPFN-CLLAHead YOLOv8n), as shown in **Figure 4**, it is a comprehensive enhancement framework that systematically improves detection accuracy while maintaining computational efficiency.

The SRC-YOLOv8n framework introduces four key innovations that synergistically address the multi-faceted challenges in apple leaf disease detection. First, the SDA-C2f (Spatial Detail Attention C2f) module replaces the original C2f backbone component, employing Space-to-Depth convolution and spatial group enhancement mechanisms to preserve critical spatial information of subtle disease features that are typically lost during traditional downsampling operations. This module was specifically chosen over alternatives like Ghost convolution or MobileNet blocks because it achieves lossless spatial information preservation through space-to-depth transformation, which is crucial for detecting small lesions and subtle disease boundaries in apple leaves. Second, the RepGFPN (Reparameterized Generalized Feature Pyramid Network) module enhances the neck architecture through a novel reparameterization strategy that optimizes multi-scale feature fusion while reducing computational overhead during inference. Unlike traditional FPN or BiFPN approaches, RepGFPN employs training-inference decoupling that maintains rich multi-branch features during training while converting to efficient single-branch operations during inference, making it particularly suitable for lightweight deployment scenarios. Third, the CLLAHead (Cross-Level Local Attention Head) module redesigns the detection head by incorporating cross-level attention mechanisms that enable effective information interaction between different scale layers, particularly enhancing the representation capability for medium-scale disease features. This design addresses the limitation of traditional detection heads that process each scale independently, and the local attention window strategy (2×2) provides an optimal balance between computational efficiency and feature interaction capability. Finally, the Inner-IoU loss function replaces traditional IoU loss, utilizing auxiliary bounding boxes with adaptive scaling factors to accelerate convergence and improve localization accuracy for complex disease regions with irregular shapes.

#### 3.3.1 SDA-C2f: achieving spatial information preservation and feature enhancement for apple leaf disease detection

In apple leaf disease detection tasks, diseased regions often exhibit subtle texture variations, irregular spot distributions, and blurred boundary features (Wang et al., 2021). Traditional YOLOv8n models face two main challenges when processing such complex scenarios: first, conventional downsampling operations tend to lose critical detail information from diseased regions; second, models struggle to effectively focus on disease feature regions while ignoring background interference. To address these issues, this study proposes the SDA-C2f (Spatial Detail Attention C2f) module to replace the original C2f module, enhancing the model’s precise recognition capability for apple leaf diseases.

Apple leaf disease visual characteristics typically manifest as spots, streaks, discoloration, and other subtle changes on leaf surfaces. These disease features occupy relatively small pixel regions in images, and their boundaries with healthy leaf tissue are often unclear. In traditional convolutional neural networks, as network depth increases, feature map spatial resolution continuously decreases. While this information compression process facilitates high-level semantic feature extraction, it inevitably causes loss of detailed information from diseased regions. Particularly in lightweight networks like YOLOv8n, aggressive downsampling strategies adopted for computational efficiency are more prone to missing small target disease features.

The SDA-C2f module addresses these issues by integrating two core components: SPD-Conv (Space-to-Depth Convolution) and SpatialGroupEnhance. SPD-Conv adopts a space-to-depth transformation strategy, converting traditional lossy downsampling operations into lossless feature reorganization processes (Zhu et al., 2025). Specifically, given an input feature map $\;\text{X}\in {\mathbb{R}}^{S\times S\times C_{1}}$, where *S* represents the spatial dimension (height and width) of the feature map, *C*_1_ denotes the number of input channels, SPD-Conv decomposes it into scale^2^ sub-feature maps through a sliding window approach with stride equal to scale:

(1) $$f_{x,y}=\{\text{X}(i+x,j+y)|i\text{ mod scale}=0,j\text{ mod scale}=0\}$$

where x,y∈{0, 1,…,scale−1} represent the offset indices within each window, and i,j are spatial coordinate indices of the original feature map. These sub-feature maps are concatenated along the channel dimension to obtain the transformed feature map:

(2) $$X^{\prime }=\text{Concat}(f_{0,0},f_{1,0},f_{0,1},\ldots ,f_{\text{scale}-1,\text{scale}-1})$$

The final output feature map $X^{\prime }\in {\mathbb{R}}^{S/\text{scale}\times S/\text{scale}\times {\text{scale}}^{2}C_{1}}\;$ ensures complete preservation of every pixel in the original feature map, avoiding asymmetric information loss in traditional strided convolutions. For our implementation, scale = 2, resulting in spatial dimension reduction by half while channel dimension increases by a factor of 4.

Building upon SPD-Conv’s complete spatial information preservation, the SpatialGroupEnhance module further introduces a grouped spatial attention mechanism to enhance diseased feature regions (Zhang et al., 2024). Given input feature map $\;X\in {\mathbb{R}}^{B\times C\times H\times W}$, where *B* is the batch size, *C* is the number of channels, *H* and *W* are height and width respectively, this module first reshapes it to $X^{\prime }\in {\mathbb{R}}^{B\times G\times \frac{C}{G}\times H\times W}$, where *G* is the number of groups. For each group  g∈{1,2,…,G}, spatial attention weights are computed as:

(3) $$\alpha_{g}=\sigma (W_{g}\cdot \text{Norm}(\text{GAP}({X^{\prime }}_{g}⊙{X^{\prime }}_{g}))+b_{g})$$

where GAP(·) denotes global average pooling operation that aggregates spatial information, Norm(·) represents batch normalization for feature standardization, *σ*(·) is the Sigmoid activation function producing attention weights in range [0,1], ⊙ denotes element-wise multiplication, and $W_{g}\in {\mathbb{R}}^{C/G\times C/G}$, $b_{g}\in {\mathbb{R}}^{C/G}$ are learnable weight matrix and bias vector respectively for group *g*. The final output is:

(4) $$Y=X^{\prime }⊙\alpha$$

where $\alpha =[\alpha_{1},\alpha_{2},\ldots ,\alpha_{G}]$ represents the concatenated attention weights across all groups. By adaptively adjusting feature response intensities at different spatial locations, SpatialGroupEnhance effectively suppresses background noise and highlights diseased feature regions, thereby improving the model’s perception capability for disease targets.

**Figure 5:**
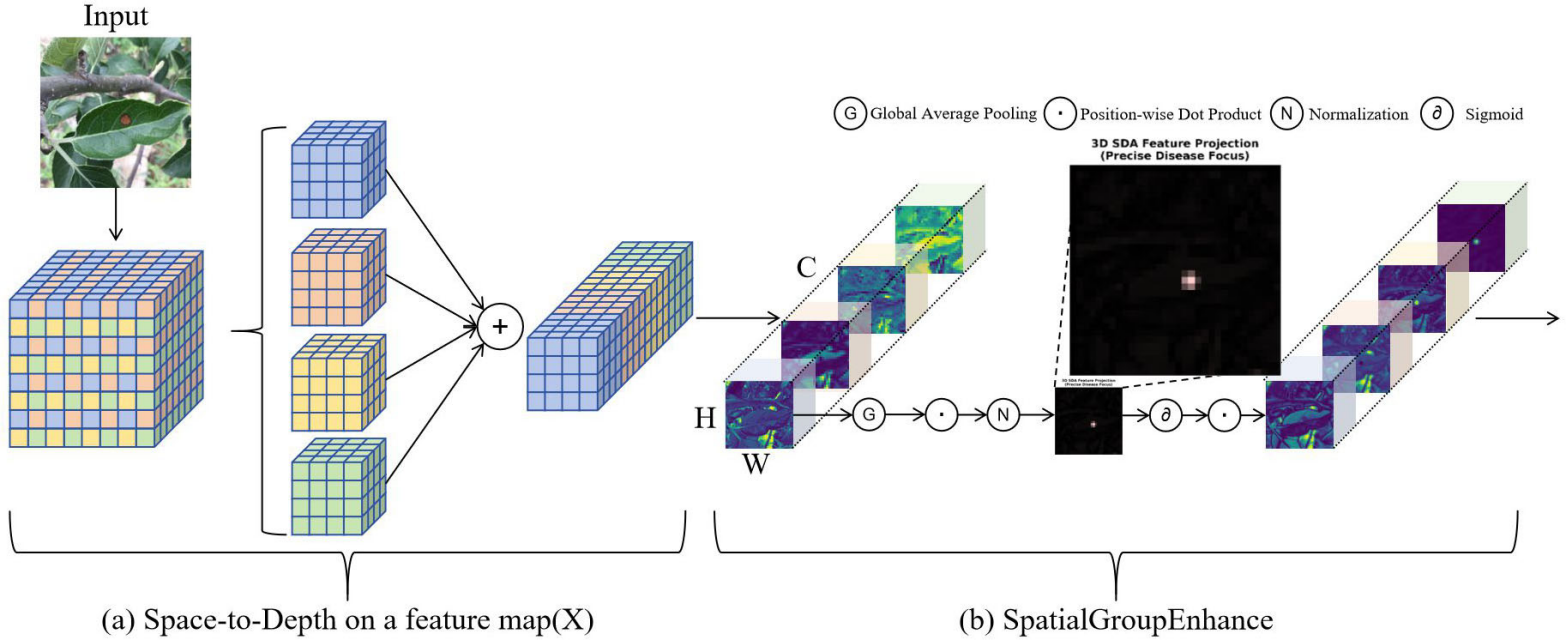
The overall architecture of the SDA-C2f module. **(a)** Left: Space-to-Depth transformation process converting spatial dimensions (H×W) to channel dimensions (C×scale^2^) while preserving all pixel information through lossless reorganization. **(b)** Right: SpatialGroupEnhance mechanism applying grouped spatial attention with global average pooling, normalization, and sigmoid activation to enhance disease-relevant features while suppressing background noise. The module integrates both components to achieve spatial detail preservation crucial for detecting subtle disease symptoms.

As shown in **Figure 5**, the overall architecture of the SDA-C2f module adopts a serial bottleneck structure similar to C2f, but replaces traditional bottleneck modules with enhanced bottleneck modules integrating SPD-Conv and SpatialGroupEnhance. This design maintains the model’s lightweight characteristics while significantly improving feature extraction accuracy and robustness. Compared to traditional C2f modules, SDA-C2f better preserves critical detail information when processing apple leaf diseases with subtle visual features and achieves precise localization of diseased regions through attention mechanisms.

#### 3.3.2 RepGFPN: efficient multi-scale feature fusion for apple leaf disease detection

In the field of plant phenomics, accurate detection of apple leaf diseases presents unique challenges due to the complex visual characteristics of diseased leaves, including irregular lesion boundaries, varying disease severities, and diverse symptom presentations across different growth stages (Liu and Wang, 2021). In YOLOv8, the neck architecture primarily relies on traditional Feature Pyramid Networks (FPNs) with simple concatenation operations for multi-scale feature fusion (Lin et al., 2017). While this approach provides basic feature integration, it faces significant limitations when dealing with complex apple leaf disease detection scenarios.

**Figure 6:**
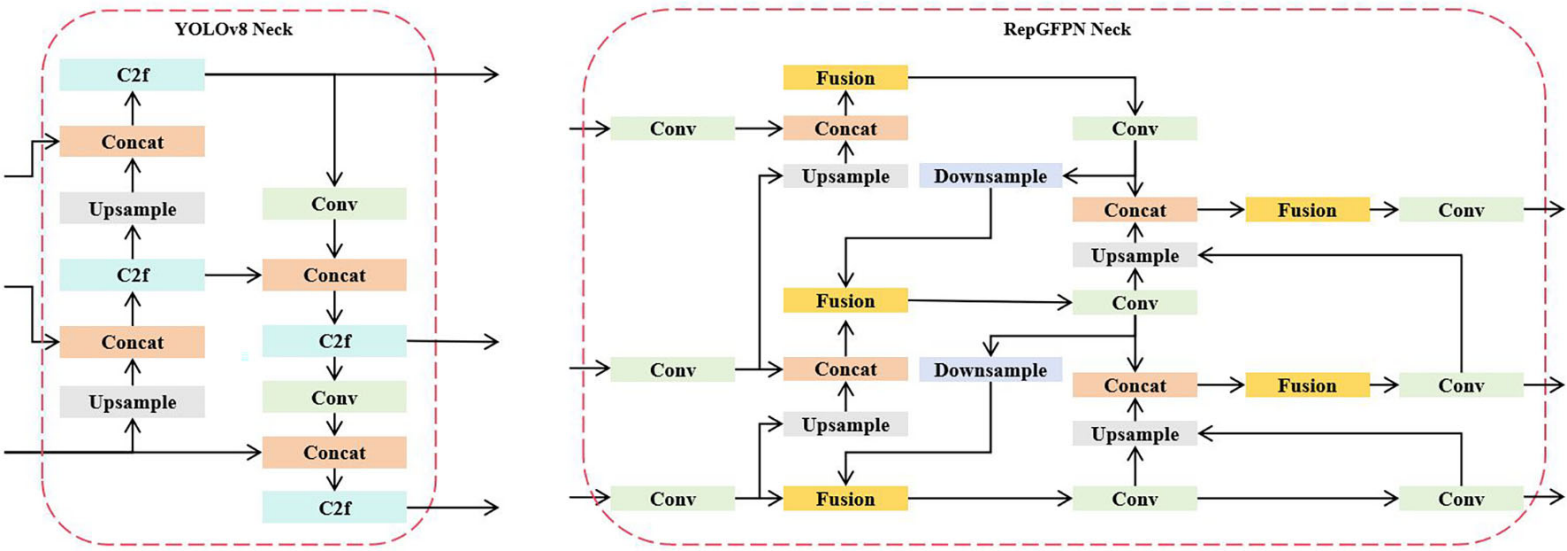
Architecture comparison between YOLOv8 neck and RepGFPN neck networks. The left side shows the traditional YOLOv8 neck with simple concatenation and upsampling operations, while the right side illustrates the proposed RepGFPN neck featuring fusion blocks, asymmetric upsampling/downsampling pathways, and enhanced multi-scale feature integration. The Rep 3×3 module enables training-inference decoupling through structural reparameterization.

To address these limitations, we introduce the Reparameterized Generalized Feature Pyramid Network (RepGFPN), a reparameterized feature pyramid architecture specifically designed to enhance multi-scale feature representation (Chu et al., 2024). The framework is shown in **Figure 6**. The RepGFPN module serves as the neck component, optimizing the fusion of high-level semantic features with low-level spatial details crucial for identifying disease-specific patterns in plant imagery. The selection of RepGFPN over alternative architectures such as BiFPN or PAFPN is motivated by its unique reparameterization strategy that decouples training and inference structures, enabling the model to maintain rich multi-branch feature representations during training while achieving efficient single-branch inference, thereby optimizing the accuracy-efficiency trade-off critical for practical agricultural deployment.

The core innovation lies in the Fusion Block, which integrates multiple input feature maps through an orchestrated sequence of operations. Unlike traditional approaches that simply concatenate features from different scales, RepGFPN employs a multi-branch fusion strategy where input features undergo parallel processing through 1×1 convolutions for channel alignment, followed by a centralized concatenation operation that preserves spatial information while enabling efficient channel-wise feature integration.

The Fusion Block incorporates a novel Simplify Rep 3×3 module that serves as the backbone of the feature transformation process. This module implements a reparameterization strategy during training and inference phases, where the training phase utilizes a multi-branch structure consisting of 3×3 convolutions, 1×1 convolutions, and batch normalization layers to capture diverse feature patterns essential for disease detection. The mathematical formulation of this process can be expressed as:

(5) $$F_{\text{rep}}=W_{3\times 3}*F_{\text{input}}+W_{1\times 1}*F_{\text{input}}+F_{\text{identity}}$$

where W_3×3_ and W_1×1_ are the convolutional kernel weights for 3×3 and 1×1 branches respectively, ∗ denotes the convolution operation, and F_identity_ represents the identity mapping. The multiple pathways enable comprehensive feature extraction. During inference, these branches are reparameterized into a single 3×3 convolution kernel through weight merging:

(6) $$F_{\text{rep}}=W_{\text{merged}}*F_{\text{input}}$$

where W_merged_ is the equivalent merged kernel obtained by converting the 1×1 kernel to 3×3 format through zero-padding and then adding all branch weights together, significantly reducing computational overhead while maintaining feature representation quality.

The RepGFPN Neck architecture implements a hierarchical feature fusion strategy that processes features from three different scale levels through a designed information flow. The top-level features undergo convolution operations followed by upsampling to provide high-level semantic information, while mid-level features are processed through fusion blocks that integrate both upsampled high-level features and lateral connections from the backbone. The bottom-level processing incorporates downsampling operations that capture fine-grained spatial details crucial for identifying small disease lesions on apple leaves (Zhang et al., 2025). A key innovation in RepGFPN is the asymmetric processing of upsampling and downsampling operations within the neck structure. RepGFPN selectively retains essential downsampling pathways that effectively aggregate semantic information from higher-level features while eliminating redundant upsampling operations.

The channel configuration strategy in RepGFPN adopts a scale-adaptive approach that allocates different channel dimensions to different feature pyramid levels. This design addresses the computational imbalance inherent in traditional FPN architectures where identical channel dimensions across all scales lead to disproportionate computational loads. The optimal channel allocation follows the formula *C_i_* = *C*_base_ × 2*^i^*^−1^, where *i* is the pyramid level index, *C*_base_ is the base channel number, smaller scale feature maps utilize fewer channels for efficient processing of high-resolution spatial information, while larger scale feature maps employ more channels to capture complex semantic relationships essential for disease classification.

The overall feature fusion process in RepGFPN integrates multiple feature pathways through a comprehensive mathematical formulation. After concatenating features from different pathways (upsampling, lateral, and downsampling), the fused features undergo processing through the reparameterized convolution structure:

(7) $$F_{\text{fused}}^{\left(l\right)}=\text{RepConv}(\text{Concat}(F_{\text{up}}^{\left(l\right)},F_{\text{lateral}}^{\left(l\right)},F_{\text{down}}^{\left(l\right)}))$$

where *l* denotes the pyramid level, RepConv(·) represents the simplified Rep 3×3 module described earlier, which applies the reparameterized convolution operation, Concat(·) is the channel-wise concatenation operation, and ${\text{F}}_{\text{up}}^{\left(l\right)}$, ${\text{F}}_{\text{lateral}}^{\left(l\right)}$, ${\text{F}}_{\text{down}}^{\left(l\right)}$ represent the upsampled features from higher levels, lateral features from the backbone at the same level, and downsampled features from lower levels respectively. This formulation ensures that each pyramid level receives comprehensive feature information while maintaining computational efficiency through the simplified inference structure.

**Figure 7:**
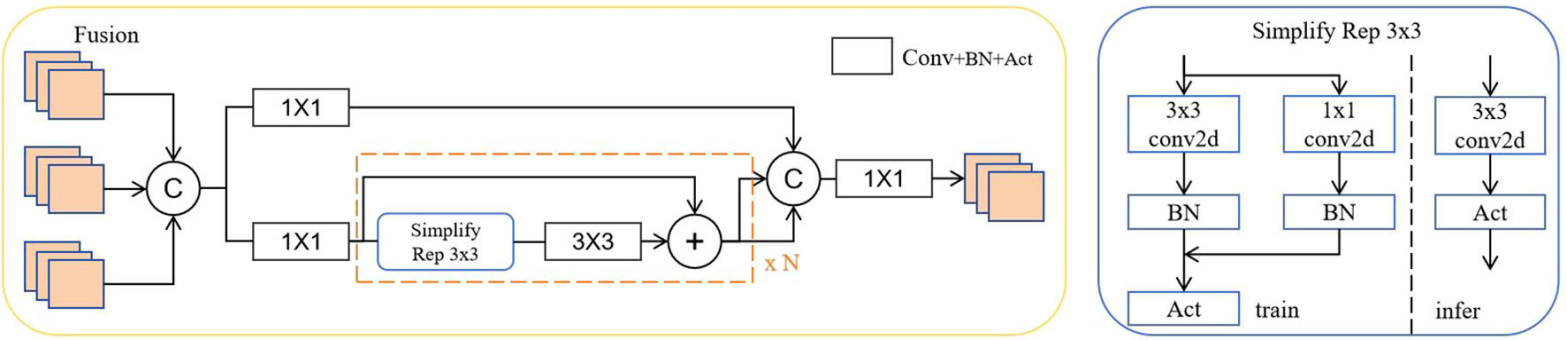
RepGFPN-fusion module for multi-scale feature integration. The network employs a dual-path architecture with feature concatenation and residual connections to enhance information flow. Multiple 1×1 convolutions align channel dimensions before the Rep 3×3 module performs feature transformation. The blocks enable efficient feature transformation while maintaining spatial coherence across different scales.

In the Fusion block shown in **Figure 7**, we can see repeated structural units composed of multiple 1×1 convolutions and a 3×3 convolution, which are usually followed by batch normalization (BN) and activation function (Act). This composite structure is different during training and inference, achieved through the “simplified Rep 3×3” structure, which uses multi-branch convolutions during training and single-branch convolution during inference to improve efficiency.

#### 3.3.3 CLLAHead: enhancing multi-scale feature fusion through cross-level local attention for apple leaf disease detection

In apple disease detection, lesions often exhibit multi-scale distribution characteristics, ranging from small spots to large affected areas. Different scales of disease features require models with strong multi-scale feature fusion capabilities. Although traditional YOLOv8 detection heads employ multi-scale detection strategies, they lack effective information interaction between different scale layers, resulting in insufficient representation capability for medium-scale disease features (Gao et al., 2024).

**Figure 8:**
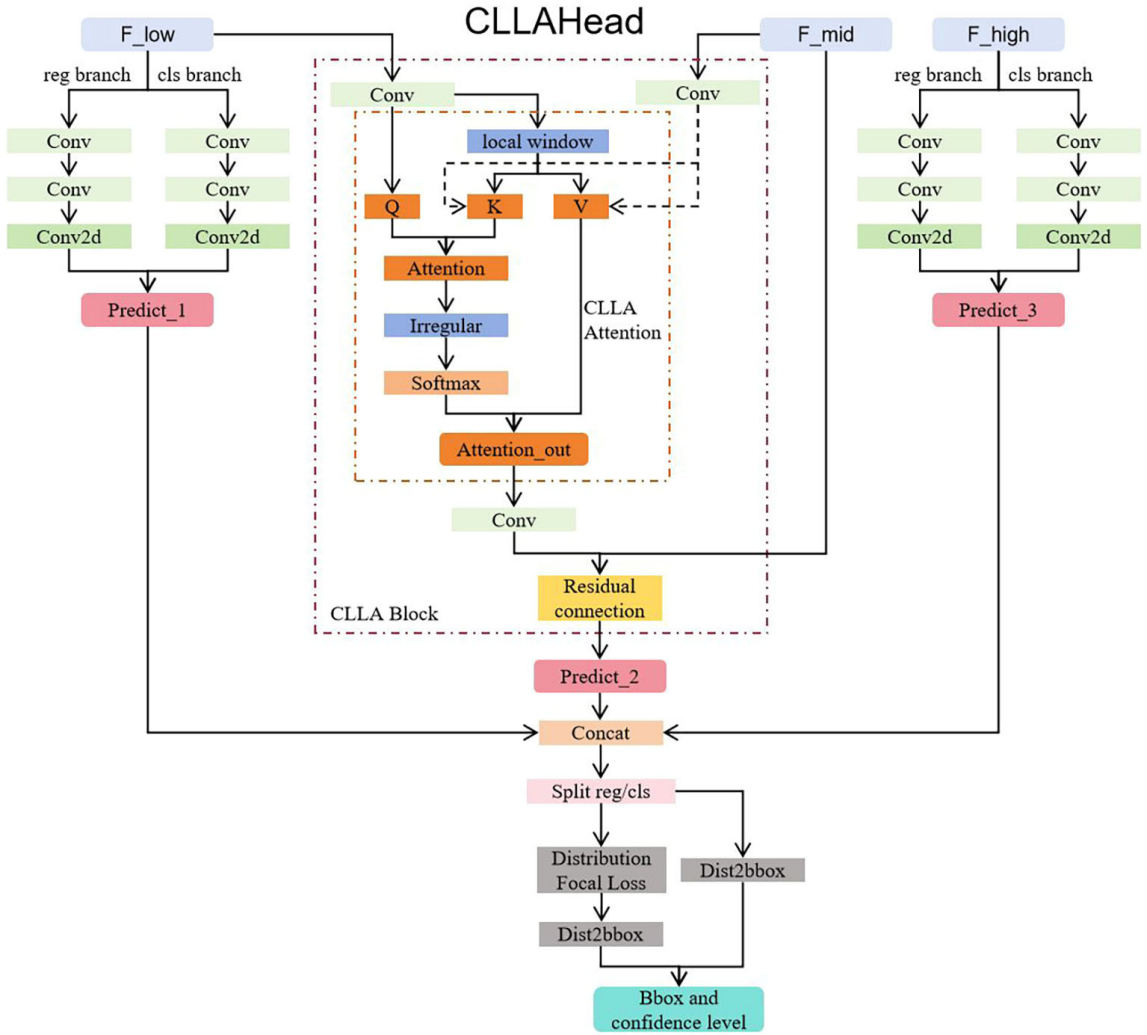
CLLAHead architecture with cross-level local attention mechanism. The network processes three pyramid levels (P: 80×80×256, P: 40×40×512, P: 20×20×512). The P layer uses the CLLA module for multi-scale feature fusion through cross-level attention between P and P features, while P and P layers employ traditional convolutional detection branches. DFL (Distribution Focal Loss) modules are applied for refined bounding box regression.

To address this issue, we designed the Cross-Level Local Attention Head (CLLAHead) to replace the original YOLOv8 detection head. As shown in **Figure 8**, CLLAHead is an innovative object detection head network architecture specifically designed to enhance multi-scale feature fusion capabilities (Li et al., 2024a; Liu et al., 2020). This architecture effectively addresses the insufficient feature representation problem in traditional detection heads when processing multi-scale targets by introducing cross-level local attention mechanisms. The motivation for adopting CLLAHead stems from the observation that apple leaf diseases often manifest at medium scales (40×40 feature level), where standard detection heads process each scale independently without cross-scale information exchange, leading to suboptimal feature representation for overlapping or multi-scale lesion patterns.

The core concept of CLLAHead is to achieve effective interaction between different scale feature layers through attention mechanisms while maintaining computational efficiency. CLLAHead adopts a three-layer detection structure corresponding to different feature scales, where we denote the three detection layers as *P*_3_, *P*_4_, and *P*_5_ following the standard FPN notation:

(8) $$\text{P}=\{P_{3},P_{4}P_{5}\}=\{\begin{array}{ll} \text{Cat}(\text{cv}2_{3}({\text{F}}_{3}),{cv3}_{3}({\text{F}}_{3})) & \text{for }P_{3}\text{ layer}\\ \text{CLLABlock}({\text{F}}_{3},{\text{F}}_{4}) & \text{for }P_{4}\text{ layer}\\ \text{Cat}(\text{cv}2_{5}({\text{F}}_{5}),{cv3}_{5}({\text{F}}_{5})) & \text{for }P_{5}\text{ layer} \end{array}$$

where F_3_, F_4_, F_5_ represent the input feature maps from the backbone network at different scales with spatial resolutions of 80×80, 40×40, and 20×20 respectively, cv2*_i_*(·) and cv3*_i_*(·) are convolutional branches for bounding box regression and classification at scale *i*, and Cat(·) denotes channel-wise concatenation. Specifically, this detection head adopts a hybrid detection strategy: P layer (80×80×256): Maintains traditional convolutional detection branches for small-scale feature processing. P layer (40×40×512): Introduces cross-level attention module for feature interaction and fusion between P and P layers, specifically targeting medium-scale disease detection. P layer (20×20×512): Maintains traditional convolutional detection branches for large-scale feature processing. This design fully considers the distribution characteristics of apple leaf diseases at medium scales, enhancing the feature representation capability of the P layer and improving the model’s detection accuracy for medium-sized lesions.

The CLLA module (Cross-Level Local Attention) is the core innovation of CLLAHead, achieving cross-level information fusion by computing local correlations between different scale feature maps. The design draws inspiration from self-attention mechanisms but is optimized for the characteristics of object detection tasks (Wu et al., 2024; Lee et al., 2024; Yang et al., 2025).

(9) $$\text{CLLA}(X_{1},X_{2})=\frac{1}{2}(V\cdot \text{Attention}(Q,K)+X_{2})$$

where Q = Linear*_q_*(X_2_) ∈ R*^B^*^×^*^H^*^2×^*^W^*^2×1×^*^C^* is the query features derived from the current scale (*P*_4_), $K={\text{Linear}}_{k}(X_{1}^{\text{local}})\in {\mathbb{R}}^{B\times H_{2}\times W_{2}\times R^{2}\times C}$ is the key features from local regions of the adjacent scale $(P_{3}),$ $V={\text{Linear}}_{v}(X_{1}^{\text{local}})\in {\mathbb{R}}^{B\times H_{2}\times W_{2}\times R^{2}\times C}$ is the value features, *B* is batch size, *H*_2_ and *W*_2_ are spatial dimensions of *P*_4_ layer, *R* is the local sampling range, and *C* is the channel dimension (set to 256 after channel alignment).

The module first uniformly adjusts the channel dimensions of P and P feature maps to 256 through 1×1 convolutions, ensuring feature dimension consistency. For input feature map $X_{1}\in {\mathbb{R}}^{B\times H_{1}mesW_{1}\times C_{1}}$ (from P layer), CLLA adopts a sliding window strategy to extract local features:

(10) $$X_{1}^{\text{local}}[i,j]=X_{1}[\text{pad }:,i::2,j::2][:W_{2},:H_{2}],\;i,j\in [0,R)$$

where *R* is the sampling range (typically set to 2), pad = ⌊*R/*2⌋ − 1 is the padding size to ensure proper alignment, and the slicing operation *i*:: 2 represents sampling with stride 2 starting from position *i*.

To reduce the computational complexity of global attention, the CLLA module introduces a local window mechanism, restricting attention computation within 2×2 local windows. During attention computation, the module employs an innovative irregularity calculation method to enhance the discriminative capability of attention weights:

(11) $$\text{dots}=\frac{1}{RC}\sum_{d=1}^{C}Q\cdot K$$

(12) irr=2·Mean(dots)−dots

(13) Attention=Softmax(irr)

where dots $\in {\mathbb{R}}^{B\times H_{2}\times W_{2}\times R^{2}}$ represents the similarity scores between query and key features, and irr (irregularity) measures the deviation of each location from the mean similarity, effectively highlighting regions with distinctive features. This design can highlight features that differ significantly from surrounding regions through the irr (irregular) term, facilitating accurate localization of disease areas. This localization strategy not only significantly reduces computational overhead but also better preserves spatial locality, aligning with the spatial continuity characteristics of lesions in disease detection.

CLLABlock serves as the encapsulated implementation of the CLLA mechanism, responsible for standardizing feature maps with different channel numbers before cross-level fusion:

(14) $$\begin{array}{l} \text{CLLABlock}(X_{1},X_{2})\\ ={\text{Conv}}_{1\times 1}(\text{CLLA}({\text{Conv}}_{1\times 1}(X_{1}),{\text{Conv}}_{1\times 1}(X_{2}))) \end{array}$$

Its mathematical expression is:

(15) $$F_{\text{out}}=W_{\text{det}}\cdot \text{CLLA}(W_{1}\cdot X_{1},W_{2}\cdot X_{2})+b_{\text{det}}$$

where $W_{\text{det}}\in {\mathbb{R}}^{C_{\text{out}}\times C}$, $W_{1}\in {\mathbb{R}}^{C\times C_{1}}$, $W_{2}\in {\mathbb{R}}^{C\times C_{2}}$ are convolution kernel parameter matrices for output projection and input mapping respectively, and $b_{\text{det}}\in {\mathbb{R}}^{C_{\text{out}}}$ is the bias vector. To maintain feature stability and avoid gradient vanishing problems, the CLLA module employs residual connections to fuse attention output with original P feature maps, with final output mapped to the required number of channels for detection through 1×1 convolution.

The DFL module (Distribution-Based Focal Loss) is based on generalized focal loss theory, improving localization accuracy by learning the distribution characteristics of bounding box regression (Li et al., 2020). Its core idea is to transform traditional point estimation into distribution estimation:

(16) $$L_{\text{DFL}}(S_{i},S_{i+1})=-((y_{i+1}-y)\text{log }(S_{i})+(y-y_{i})\text{log }(S_{i+1}))$$

where $S_{i}$ and $S_{i+1}$ are predicted probabilities of two adjacent discrete positions in the distribution, *y* is the ground truth label value, and $y_{i}$, $y_{i+1}\;$ are the discrete position values satisfying $\;y_{i}\leq y\leq y_{i+1}$.

The forward propagation process of the DFL module can be expressed as:

(17) DFL(x)=Conv1D(Softmax(Reshape(x)))

where $\;\text{x}\in {\mathbb{R}}^{B\times (4\times c_{1})}$ is the input regression prediction with *c*_1_ discrete bins for each of the 4 box coordinates, Reshape(·) converts the input to shape $\;{\mathbb{R}}^{B\times 4\times c_{1}}$, Softmax(·) normalizes the distribution, and the convolution kernel weights are initialized as: $\;W_{DFL}={\left[0,\;1,\;2,\ldots ,c_{1}-1\right]}^{T}\in {\mathbb{R}}^{c_{1}\times 1}$, which performs weighted sum to obtain the expected value of the distribution.

The overall architecture of CLLAHead maintains the basic framework of the YOLOv8 detection head, including the design of bounding box regression and classification branches. For P and P layers, the detection head continues to use traditional convolutional branches, with each branch containing two 3×3 convolutional layers for feature extraction, finally outputting detection results through 1×1 convolution. The P layer output comes from enhanced features of the CLLA module.

The final output decoding process generates results through the following steps:

(18) $$\begin{array}{l} X_{\text{cat}}=\text{Concat}([P_{i}.\text{view}(B,N_{o},-1)\\ \text{ for }i\in \{0,\;1,\;2\}],\text{dim}=2) \end{array}$$

(19) dbox=dist2bbox(DFL(box),anchors)×strides

(20) Y=Concat(dbox,σ(cls))

where *N_o_* is the number of output channels per location (4 for box coordinates plus number of classes), box and cls represent the box regression and classification outputs respectively, dist2bbox(·) converts the distribution to box coordinates, anchors are the predefined anchor points, strides are the downsampling factors for each layer ([8, 16, 32] for P, P, P), and *σ*(·) is the Sigmoid activation function used for class probability normalization. Detection results from three scale layers are concatenated and decoded using Distribution Focal Loss (DFL) for bounding boxes, transforming regression prediction from discrete classification problems to continuous regression problems, improving bounding box localization accuracy.

CLLAHead employs an adaptive bias initialization strategy to improve training stability: bias_box_ = 1.0, ${\text{bias}}_{\text{cls}}=\text{log }(\frac{5}{N_{c}\cdot {\left(640/s\right)}^{2}})$, where *N_c_* is the number of classes (7 in our case), and *s* is the stride of the corresponding layer (8, 16, or 32). This initialization strategy, based on prior statistical information about object frequency and scale distribution, facilitates rapid network convergence by providing reasonable initial predictions.

#### 3.3.4 Inner-IoU: enhancing bounding box regression for apple leaf disease detection

In YOLOv8n, bounding box regression is a critical factor affecting the accuracy of apple leaf disease detection. Traditional IoU loss functions suffer from slow convergence and weak generalization capabilities (Gevorgyan, 2022), particularly when dealing with complex leaf disease symptoms with irregular boundaries. To address these limitations, we introduce the Inner-IoU loss function to replace traditional IoU loss, thereby improving the accuracy and efficiency of apple leaf disease detection. The core idea of Inner-IoU is to compute the loss through auxiliary bounding boxes (Zhang et al., 2023), optimizing the bounding box regression process. Unlike traditional IoU loss, Inner-IoU employs a scaling factor ratio to control the scale of auxiliary bounding boxes, enabling adaptive adjustment according to different regression samples. For high IoU samples, smaller auxiliary bounding boxes can accelerate convergence, while larger auxiliary bounding boxes are more suitable for low IoU samples. The computation process of Inner-IoU is as follows: Given predicted box *B*_1_ = (*x*_1_*,y*_1_*,w*_1_*,h*_1_) and ground truth box *B*_2_ = (*x*_2_*,y*_2_*,w*_2_*,h*_2_), where (*x_i_,y_i_*) represent the center coordinates and (*w_i_,h_i_*) represent the width and height of the bounding box respectively, we construct auxiliary bounding boxes through the scaling factor ratio:

(21) $$B_{1}^{'}=(x_{1},y_{1},w_{1}\times \text{ratio},h_{1}\times \text{ratio})$$

(22) $$B_{2}^{'}=(x_{2},y_{2},w_{2}\times \text{ratio},h_{2}\times \text{ratio})$$

Subsequently, the Inner-IoU is calculated as:

(23) $$\text{Inner}-\text{IoU}=\frac{Area(B_{1}^{'}\cap B_{2}^{'})}{Area(B_{1}^{'}\cup B_{2}^{'})}$$

First, we define the coordinates of the scaled bounding boxes. Let *r* = ratio:

(24) $$x_{\text{min}}^{1}=x_{1}-\frac{w_{1}\times r}{2},\;x_{\text{max}}^{1}=x_{1}+\frac{w_{1}\times r}{2}$$

(25) $$y_{\text{min}}^{1}=y_{1}-\frac{h_{1}\times r}{2},\;y_{\text{max}}^{1}=y_{1}+\frac{h_{1}\times r}{2}$$

(26) $$x_{\text{min}}^{2}=x_{2}-\frac{w_{2}\times r}{2},\;x_{\text{max}}^{2}=x_{2}+\frac{w_{2}\times r}{2}$$

(27) $$y_{\text{min}}^{2}=y_{2}-\frac{h_{2}\times r}{2},\;y_{\text{max}}^{2}=y_{2}+\frac{h_{2}\times r}{2}$$

The intersection and union areas are defined as:

(28) $$\begin{array}{ll} \text{Area}(B_{1}^{'}\cap B_{2}^{'})= & \text{max}(0,\text{min}(x_{\text{max}}^{1},x_{\text{max}}^{2})-\text{max}(x_{\text{min}}^{1},x_{\text{min}}^{2}))\\ \times \text{max}(0,\text{min}(y_{\text{max}}^{1},y_{\text{max}}^{2})-\text{max}(y_{\text{min}}^{1},y_{\text{min}}^{2})) \end{array}$$

(29) $$\begin{array}{l} \text{Area}(B_{1}^{'}\cup B_{2}^{'})=w_{1}\times h_{1}\times r^{2}+w_{2}\times h_{2}\times r^{2}-\text{Area}(B_{1}^{'}\\ \cap B_{2}^{'}) \end{array}$$

In apple leaf disease detection tasks, the advantages of Inner-IoU are primarily manifested in: (1) Through adaptive auxiliary bounding box scales, it can better handle disease regions of different sizes ranging from small spots to large affected areas; (2) It improves the model’s detection accuracy for small target diseases by providing more stable gradients during training; (3) It accelerates the convergence speed during training by adaptively adjusting the difficulty of the regression task based on the current IoU level. Experimental results demonstrate that the YOLOv8n model employing Inner-IoU loss achieves significant performance improvements in apple leaf disease detection tasks.

### 3.4 Test environment

**Tables 3** and **4** show the experimental environment and parameters, respectively. The experimental platform consisted of an NVIDIA GeForce RTX 3060 graphics card paired with an Intel(R) Core(TM) i5-12400F processor, operating under Windows 10 with PyTorch serving as the deep learning computational framework (Paszke, 2019). To ensure reproducibility and consistency throughout all experiments, standardized model parameters were maintained across all network configurations.

**Table 3:** Experimental environment.

Environment	Configuration
Operating system	Windows 10
CPU	Intel(R) Core(TM) i5-12400F
GPU	NVIDIA GeForce RTX 3060
RAM	16GB
Programming language	Python 3.8.20
Deep learning framework	Pytorch 1.13.1
CUDA	11.6

**Table 4:** Experimental parameters.

Parameters	Setup
Input image size	640 × 640
Batch size	8
Optimizer	SGD
Learning rate	0.01
Epochs	1000

The training optimization employed stochastic gradient descent with an initial learning rate set to 0.01. To mitigate overfitting effects, a weight decay regularization coefficient of 0.0005 was applied, while the training batch size was configured to 8 samples. The training protocol incorporated a preliminary warm-up period of 3 epochs to facilitate gradual adaptation to the optimal learning rate, subsequently followed by an extensive training phase extending over 1000 epochs. All input images underwent preprocessing to achieve uniform dimensions of 640 × 640 pixels, ensuring consistent data format standardization for network input requirements.

### 3.5 Evaluate metrics

To comprehensively assess the performance of the proposed models, this research employs multiple evaluation criteria including precision, recall, mean Average Precision at IoU threshold 0.5 (mAP50), and F1-score. These metrics provide a thorough analysis of detection accuracy from different perspectives.

Precision (Powers, 2020) quantifies the proportion of correctly predicted positive instances among all predicted positive samples, while recall (Sokolova and Lapalme, 2009) measures the fraction of actual positive samples that were successfully identified. The mathematical formulations for these metrics are expressed as:

(30) $$\text{Precision}=\frac{TP}{TP+FP}$$

(31) $$\text{Recall}=\frac{TP}{TP+FN}$$

where *TP* (True Positive), *FP* (False Positive), and *FN* (False Negative) represent the corresponding classification outcomes.

Given that precision and recall individually provide limited insight into overall detection performance, additional metrics are incorporated for comprehensive evaluation. The mAP50 metric represents the mean Average Precision (Henderson and Ferrari, 2016) calculated at an Intersection over Union (IoU) (Rezatofighi et al., 2019) threshold of 0.5, which is derived from the IoU between predicted and ground truth bounding boxes. The Average Precision (AP50) is computed by integrating the area under the precision-recall curve across the entire recall range [0,1] with IoU threshold fixed at 0.5.

The AP50 and mAP50 are formulated as:

(32) $$\text{AP}50=\int_{0}^{1}P(r)\;dr$$

(33) $$\text{mAP}50=\frac{1}{n}\sum_{i=1}^{n}\text{AP}50_{i}$$

where *P*(*r*) is the precision as a function of recall *r*, and *n* denotes the total number of object classes in the dataset (7 disease classes in our case).

The F1-score (Chicco and Jurman, 2020) provides a harmonic mean of precision and recall, offering a balanced assessment of model performance:

(34) $$\text{F}1-\text{score}=2\times \frac{\text{Precision}\times \text{Recall}}{\text{Precision}+\text{Recall}}$$

Beyond accuracy metrics, computational efficiency is evaluated through model complexity indicators including model size (in MB), parameter count (in millions), inference time per image, and frames per second (FPS) to assess the practical deployment feasibility of the proposed approaches.

## 4 Results

### 4.1 Experimental setup and dataset configuration

To address the concerns regarding experimental reproducibility and fairness in comparative evaluation, we provide detailed clarification of our experimental configuration. All experiments reported in this study, including ablation studies (Section 4.2), module comparisons (Sections 4.3-4.6), and state-of-the-art comparisons (Section 4.7), were conducted on our combined Apple-Disease-Detection Dataset constructed from Plant-Pathology-2021-FGVC8 and AppleLeaf9 datasets as described in Section 3.2.

The dataset partitioning follows a rigorous protocol to ensure fair evaluation: the original 3,555 images were first split into training (80%), validation (10%), and test (10%) sets before any data augmentation was applied. This separation ensures that no augmented versions of test images appear in the training or validation sets, thereby preventing data leakage. The augmentation techniques (horizontal flipping, vertical flipping, translation, contrast adjustment, and brightness adjustment) were only applied to the training and validation sets to enhance model generalization capability, while the test set remains entirely composed of original, unaugmented images to provide unbiased performance evaluation.

For all comparative experiments presented in **Tables 5**–**9**, we reimplemented or fine-tuned the compared methods using identical training configurations (learning rate=0.01, batch size=8, epochs=1000, image size=640×640) on our combined training set and evaluated them on our original test set. This standardized evaluation protocol ensures fair comparison across different architectures. The performance metrics reported for state-of-the-art methods in **Table 9** represent their performance on our test set after retraining, rather than results copied from original publications, thereby eliminating bias from different dataset compositions or evaluation protocols.

**Table 5:** Results of ablation experiments with metrics of mAP50, F1 score, FPS, parameter size, GFLOPs, and model size.

SDA-C2f	RepGFPN	CLLAHead	Inner-IoU	P (%)	R (%)	mAP50 (%)	mAP50-95 (%)	F1 (%)	FPS	Params (M)	GFLOPs	Size (MB)
				91.2	89.5	93.8	64.1	90.3	145.2	3.01	8.1	6.2
✔				92.1	90.2	94.3	64.8	91.1	156.7	2.84	7.6	5.8
✔	✔			92.8	91.1	94.9	65.4	91.9	148.3	2.79	7.2	5.7
✔	✔	✔		93.4	91.7	95.4	66.2	92.5	142.1	2.56	6.8	5.2
✔	✔	✔	✔	**94.1**	**92.3**	**96.1**	**67.1**	**93.2**	**138.9**	**2.51**	**6.5**	**5.1**

Bold font indicates the optimal result. All experiments were conducted on the original test set of the Apple-Disease-Detection Dataset.

**Table 6:** Comparisons of different backbone C2f network modules with metrics of mAP50, F1 score, FPS, parameter size, GFLOPs, and model size.

Models	P (%)	R (%)	mAP50 (%)	mAP50-95 (%)	F1 (%)	FPS	Params (M)	GFLOPs	Size (MB)
YOLOv8n-C2f (Reis et al., 2023)	91.2	89.5	93.8	64.1	90.3	145.2	3.01	8.1	6.2
YOLOv8n-C2f-Ghost (Han et al., 2020)	91.5	89.8	94.1	64.3	90.6	152.1	2.96	7.8	6.0
YOLOv8n-C2f-MobileNet (Howard et al., 2017)	91.8	90.1	94.2	64.5	90.9	148.7	2.91	7.9	5.9
YOLOv8n-C2f-ShuffleNet (Zhang et al., 2018)	92.0	90.3	94.4	64.6	91.1	149.3	2.89	7.7	5.8
YOLOv8n-C2f-EfficientNet (Tan and Le, 2019)	92.2	90.5	94.5	64.8	91.3	147.9	2.87	7.6	5.8
YOLOv8n-SDA-C2f	**92.1**	**90.2**	**94.3**	**64.8**	**91.1**	**156.7**	**2.84**	**7.6**	**5.8**

Bold font indicates the optimal result. All models were trained and evaluated under identical conditions on our dataset.

**Table 7:** Comparison of different feature fusion modules with metrics of mAP50, F1 score, FPS, parameter size, GFLOPs, and model size.

Models	P (%)	R (%)	mAP50 (%)	mAP50-95 (%)	F1 (%)	FPS	Params (M)	GFLOPs	Size (MB)
YOLOv8n-PANet (Reis et al., 2023)	91.2	89.5	93.8	64.1	90.3	145.2	3.01	8.1	6.2
YOLOv8n-FPN (Lin et al., 2017)	92.3	90.4	94.5	64.9	91.3	154.2	2.87	7.8	5.9
YOLOv8n-BiFPN (Tan et al., 2020)	92.4	90.6	94.6	65.1	91.5	151.8	2.91	8.0	6.0
YOLOv8n-ASFF (Liu et al., 2019)	92.6	90.8	94.7	65.2	91.7	149.5	2.95	8.2	6.1
YOLOv8n-PAFPN (Liu et al., 2018)	92.7	90.9	94.8	65.3	91.8	150.1	2.93	8.1	6.0
YOLOv8n-SDA-C2f-RepGFPN	**92.8**	**91.1**	**94.9**	**65.4**	**91.9**	**148.3**	**2.79**	**7.2**	**5.7**

Bold font indicates the optimal result. All methods were evaluated using SDA-C2f as the backbone under identical training conditions.

**Table 8:** Comparison of different detection head modules with metrics of mAP50, F1 score, FPS, parameter size, GFLOPs, and model size.

Models	P (%)	R (%)	mAP50 (%)	mAP50-95 (%)	F1 (%)	FPS	Params (M)	GFLOPs	Size (MB)
YOLOv8n-OriginalHead (Reis et al., 2023)	91.2	89.5	93.8	64.1	90.3	145.2	3.01	8.1	6.2
YOLOv8n-DecoupledHead (Qiu et al., 2023)	92.9	91.2	95.0	65.5	92.0	146.1	2.82	7.4	5.8
YOLOv8n-AuxHead (Jin et al., 2020)	93.1	91.3	95.1	65.7	92.2	143.7	2.95	7.8	6.1
YOLOv8n-LADHead (Jiang et al., 2021)	92.7	91.0	94.8	65.2	91.8	151.2	2.64	6.8	5.4
YOLOv8n-SEAMHead (Ghosh, 2024)	93.2	91.4	95.2	65.8	92.3	144.6	2.78	7.1	5.6
YOLOv8n-SDA-C2f-RepGFPN-CLLAHead	**93.4**	**91.7**	**95.4**	**66.2**	**92.5**	**142.1**	**2.56**	**6.8**	**5.2**

Bold font indicates the optimal result. All methods were evaluated using SDA-C2f backbone and RepGFPN neck under identical conditions.

**Table 9:** Comparison of different loss functions with metrics of mAP50, precision, recall, and F1 score.

Models	P (%)	R (%)	mAP50 (%)	mAP50-95 (%)	F1 (%)	FPS	Parameter size	GFLOPs	Model size
YOLOv8n-CIoU (Wang and Song, 2021)	93.4	91.7	95.4	66.2	92.5	142.1	2.56	6.8	5.2
YOLOv8n-DIoU (Zheng et al., 2020)	93.2	91.5	95.2	65.9	92.3	142.8	2.56	6.8	5.2
YOLOv8n-GIoU (Liu et al., 2022)	93.1	91.3	95.1	65.7	92.2	143.2	2.56	6.8	5.2
YOLOv8n-EIoU (Zhang et al., 2022)	93.5	91.8	95.5	66.3	92.6	141.9	2.56	6.8	5.2
YOLOv8n-WIoU (Tong et al., 2023)	93.6	91.9	95.6	66.4	92.7	141.7	2.56	6.8	5.2
YOLOv8n-SDA-C2f-RepGFPN-CLLAHead-Inner-IoU	**94.1**	**92.3**	**96.1**	**67.1**	**93.2**	**138.9**	**2.51**	**6.5**	**5.1**

Bold font indicates the optimal result. All methods were evaluated using the complete SRC-YOLOv8n architecture (SDA-C2f + RepGFPN + CLLAHead) with only the loss function varying.

### 4.2 Results of ablation experiments

To systematically evaluate the contribution of each proposed component and understand their synergistic effects, we conduct comprehensive ablation studies following a sequential integration strategy. **Table 5** presents the progressive performance improvements achieved through incremental module addition, demonstrating both the individual contribution and cumulative effect of each innovation.

#### 4.2.1 Sequential module integration analysis

The ablation study follows a carefully designed integration sequence that reflects the hierarchical information flow in the detection pipeline: backbone feature extraction (SDA-C2f) → neck feature fusion (RepGFPN) → detection head (CLLAHead) → loss optimization (Inner-IoU). This order enables systematic evaluation of how improvements in each stage contribute to overall performance.

Stage 1 - Baseline Performance: The baseline YOLOv8n model establishes our starting point with 91.2% precision, 89.5% recall, 93.8% mAP50, and 90.3% F1 score. While demonstrating competitive performance, the baseline model shows limitations in preserving spatial details during downsampling and suffers from information loss in fine-grained disease feature extraction.

Stage 2 - SDA-C2f Integration (+0.8% F1): The integration of the Spatial Detail Attention C2f module yields immediate improvements across all metrics, with F1 score increasing to 91.1% (+0.8%), mAP50 reaching 94.3% (+0.5%), and recall improving to 90.2% (+0.7%). More significantly, the module achieves 5.6% parameter reduction (from 3.01M to 2.84M) while simultaneously improving inference speed to 156.7 FPS (+7.9%). This seemingly counterintuitive improvement in both efficiency and accuracy stems from the Space-to-Depth convolution’s ability to preserve spatial information without traditional lossy downsampling operations, thereby requiring fewer parameters to achieve better feature representation. The SpatialGroupEnhance mechanism further contributes by focusing computational resources on disease relevant regions while suppressing background noise, explaining the concurrent gains in both speed and accuracy.

Stage 3 - RepGFPN Addition (+0.8% F1): Building upon the enhanced backbone features, RepGFPN integration further improves F1 score to 91.9% (+0.8% over Stage 2), with mAP50 reaching 94.9% (+0.6%). The recall metric shows particularly notable improvement to 91.1% (+0.9%), indicating better detection completeness for disease instances. The reparameterization strategy enables efficient multi-scale feature fusion, with computational cost decreasing to 7.2 GFLOPs (-5.3% from Stage 2) despite the additional fusion operations. This efficiency gain results from the training-inference decoupling mechanism, where the multi-branch structure during training is converted to streamlined single-branch convolutions during inference. The asymmetric processing of upsampling and downsampling pathways, combined with scale adaptive channel allocation, contributes to the improved recall by effectively aggregating features across different scales.

Stage 4 - CLLAHead Integration (+0.6% F1): The incorporation of Cross-Level Local Attention Head brings F1 score to 92.5% (+0.6%), with mAP50 improving to 95.4% (+0.5%). The most substantial impact appears in parameter reduction, decreasing from 2.79M to 2.56M (-8.2%), and computational cost dropping to 6.8 GFLOPs (-5.6%). The precision metric shows notable improvement to 93.4% (+0.6%), suggesting enhanced discrimination capability for disease classification. CLLAHead’s cross-level attention mechanism enables effective information interaction between feature pyramid levels, particularly benefiting medium-scale disease detection through the P4 layer enhancement. The local window attention strategy (2×2 windows) maintains computational efficiency while capturing spatial dependencies crucial for accurate disease localization.

Stage 5 - Inner-IoU Loss Optimization (+0.7% F1): The final integration of Inner-IoU loss function completes the framework, achieving 93.2% F1 score (+0.7%), 96.1% mAP50 (+0.7%), and 94.1% precision (+0.7%). The recall reaches 92.3% (+0.6%), indicating improved detection completeness. While FPS decreases slightly to 138.9 (-2.3%), this represents only a 1.6% speed reduction from Stage 4, which is acceptable given the substantial accuracy gains. The adaptive auxiliary bounding box strategy of Inner-IoU loss enables more effective optimization for disease regions with varying sizes and irregular shapes, particularly improving localization accuracy as reflected in the mAP50–95 metric increase to 67.1% (+0.9%).

#### 4.2.2 Cumulative performance analysis

Comparing the complete SRC-YOLOv8n framework against the baseline reveals substantial improvements: mAP50 increases by 2.3 percentage points (93.8% → 96.1%), F1 score improves by 2.9 percentage points (90.3% → 93.2%), and recall shows the most significant enhancement of 2.8 percentage points (89.5% → 92.3%). The recall improvement is particularly noteworthy, indicating that the model achieves better detection completeness, missing fewer disease instances—a critical characteristic for practical agricultural monitoring applications where undetected diseases can lead to crop losses.

The efficiency metrics demonstrate the framework’s lightweight nature: parameters decrease by 16.6% (3.01M → 2.51M), computational complexity reduces by 19.8% (8.1 → 6.5 GFLOPs), and model size compresses by 17.7% (6.2MB → 5.1MB). Although FPS decreases from 145.2 to 138.9 (-4.3%), the framework maintains real-time processing capability (¿30 FPS) suitable for practical deployment. This slight speed trade-off (6.3 fps reduction) is negligible compared to the substantial accuracy and efficiency gains, representing an optimal balance for resource-constrained agricultural monitoring scenarios.

#### 4.2.3 Visual comparison of detection results

**Figure 9:**
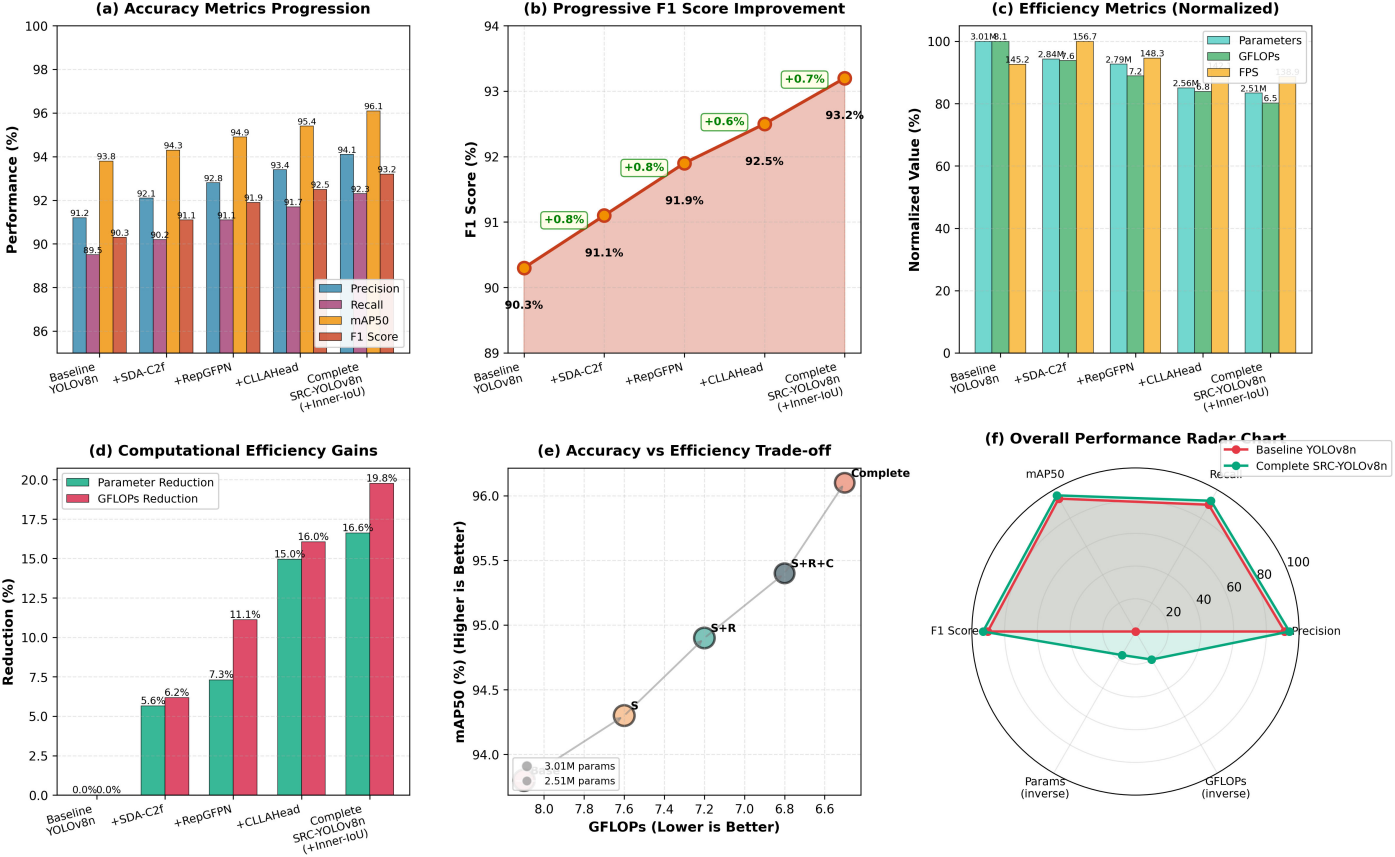
Visual comparison of detection results across ablation stages. Each row shows the same test image processed by different model configurations: **(a)** Bar chart of accuracy metrics showing improvements in precision, recall, mAP50, and F1 score with each enhancement **(b)** Line graph displaying progressive F1 score improvements from baseline 90.3% to 93.2% **(c)** Bar chart depicting normalized efficiency metrics in parameters, GFLOPs, and FPS **(d)** Bar chart on computational efficiency gains showing parameter and GFLOPs reductions **(e)** Scatter plot demonstrating accuracy versus efficiency trade-off, with improvements marked by reduced GFLOPs and increased mAP50**(f)** Radar chart comparing overall performance metrics between baseline YOLOv8n and complete SRC-YOLOv8n model enhancements. The progressive improvements in bounding box accuracy, confidence scores, and detection completeness are clearly visible, particularly for challenging cases with small lesions or complex backgrounds.

To provide intuitive understanding of each module’s contribution, **Figure 9** presents qualitative comparisons of detection results across different integration stages on representative disease samples. The visualizations demonstrate progressive improvements in localization accuracy, confidence scores, and ability to detect challenging cases such as small lesions, overlapping disease symptoms, and diseases under varying lighting conditions.

The baseline model (**Figure 9a**) occasionally produces imprecise bounding boxes or misses small disease regions. With SDA-C2f integration (**Figure 9b**), spatial detail preservation enables more accurate boundary delineation. RepGFPN addition (**Figure 9c**) improves multi-scale detection, successfully identifying disease instances across different sizes. CLLAHead integration (**Figure 9d**) refines classification confidence and reduces false positives through enhanced feature discrimination. The complete framework with Inner-IoU (**Figure 9e**) achieves optimal bounding box fitting and highest confidence scores, demonstrating the synergistic effect of all components.

### 4.3 Comparison of different backbone C2f network modules

We evaluate various backbone C2f network architectures to identify the most effective configuration for apple leaf disease detection. **Table 6** presents comprehensive comparisons with popular lightweight backbone alternatives.

The proposed SDA-C2f module demonstrates superior speed-efficiency trade-offs, achieving the highest FPS of 156.7 while maintaining competitive accuracy. Although EfficientNet-based backbone achieves marginally higher precision (92.2% vs 92.1%), SDA-C2f excels in inference speed (+5.9% over EfficientNet) and parameter efficiency. The performance differences among top methods are relatively small (F1 scores ranging from 91.1% to 91.3%), but SDA-C2f’s combination of spatial detail preservation through Space-to-Depth convolution and attention-based feature enhancement provides the optimal balance for real-time disease detection applications. Notably, while some methods show slightly higher individual accuracy metrics, SDA-C2f’s substantial speed advantage (6.4-8.0 fps faster than competitors) makes it more suitable for deployment in resource-constrained agricultural monitoring scenarios where real-time processing is essential.

### 4.4 Comparison of different feature fusion modules

We investigate various feature fusion strategies to optimize multi-scale feature integration. **Table 7** presents the comparative analysis.

RepGFPN achieves the optimal balance between detection accuracy and computational efficiency. Compared to traditional FPN (Lin et al., 2017), RepGFPN improves mAP50 by 0.4% while reducing parameters by 2.8% and GFLOPs by 7.7%. The superior performance stems from the reparameterization strategy that maintains rich multi-branch feature representations during training while converting to efficient single-branch inference. The adaptive feature aggregation mechanism with asymmetric upsampling/downsampling pathways effectively captures multi-scale disease symptoms ranging from small lesions to large infected areas. While RepGFPN shows slightly lower FPS compared to FPN (148.3 vs 154.2), the accuracy improvements (+0.4% mAP50, +0.6% F1) and significant computational efficiency gains (-2.8% parameters, -7.7% GFLOPs) justify this trade-off for deployment scenarios where model size and computational resources are constrained.

### 4.5 Comparison of different detection head modules

We evaluate various detection head architectures to determine the most suitable approach for fine-grained disease detection. **Table 8** presents the comparative analysis.

CLLAHead achieves superior performance across all accuracy metrics while maintaining competitive computational efficiency. Compared to the original detection head, CLLAHead improves mAP50 by 1.6%, F1 score by 2.2%, and reduces parameters by 15.0%. The cross-level local attention mechanism enables precise localization of disease symptoms through effective information interaction between feature pyramid levels. The P4 layer enhancement with 2×2 local attention windows particularly benefits medium-scale disease detection, which is common in apple leaf disease scenarios. Although CLLAHead shows slightly lower FPS than LADHead (Jiang et al., 2021) (142.1 vs 151.2), the substantial accuracy improvements (+0.6% mAP50, +0.7% F1) and parameter efficiency gains make it the optimal choice for applications prioritizing detection quality. The channel-level and location-level attention mechanisms effectively handle the complex visual patterns characteristic of plant diseases, explaining the superior precision (93.4%) and recall (91.7%) performance.

### 4.6 Comparison of different loss functions

We compare different loss functions to evaluate their impact on bounding box regression accuracy. **Table 9** presents the comprehensive comparison.

The Inner-IoU loss function demonstrates superior performance across all evaluation metrics. Compared to the widely-used CIoU loss (Wang and Song, 2021), Inner-IoU achieves 0.7% improvements in both precision and F1 score, with mAP50 increasing by 0.7% and mAP50–95 improving by 0.9%. The adaptive auxiliary bounding box strategy enables more effective optimization for disease regions with varying sizes and irregular shapes, which are characteristic of apple leaf diseases. The scaling factor mechanism (ratio parameter) allows the loss function to adapt to different regression scenarios: smaller auxiliary boxes for high-IoU samples accelerate convergence, while larger auxiliary boxes for low-IoU samples improve localization accuracy. This adaptive behavior particularly benefits the detection of irregular lesion boundaries common in diseases like alternaria blotch and brown spot. The slight FPS decrease (2.3% compared to CIoU) is acceptable given the substantial accuracy gains, and the framework maintains real-time processing capability above 30 FPS threshold required for practical deployment.

### 4.7 Comparison with state-of-the-art models

We compare our proposed SRC-YOLOv8n with state-of-the-art object detection models to validate its effectiveness for apple leaf disease detection. All compared models were retrained and evaluated under identical conditions using our Apple-Disease-Detection Dataset to ensure fair comparison. **Table 10** presents the comprehensive comparison results.

**Table 10:** Comparison with state-of-the-art models on the Apple-Disease-Detection Dataset (combined Plant-Pathology-2021-FGVC8 and AppleLeaf9).

Models	P (%)	R (%)	mAP50 (%)	mAP50-95 (%)	F1 (%)	FPS	Params (M)	GFLOPs	Size (MB)
Faster R-CNN (Ren et al., 2015)	89.2	87.1	91.3	59.8	88.1	23.4	41.8	207.2	158.4
SSD-MobileNet (Liu et al., 2016)	88.7	86.9	90.8	58.9	87.8	87.2	24.3	45.6	92.1
YOLOv5s (Jocher et al., 2022)	90.8	88.5	92.7	62.1	89.6	125.3	7.23	16.4	14.1
YOLOv7-tiny (Wang et al., 2023)	91.3	89.1	93.2	63.2	90.2	134.7	6.02	13.2	11.7
YOLOv8n (Reis et al., 2023)	91.2	89.5	93.8	64.1	90.3	145.2	3.01	8.1	6.2
RT-DETR-tiny (Zhao et al., 2024)	91.6	89.8	94.1	64.5	90.7	98.7	9.87	21.3	18.2
YOLOv10n (Wang et al., 2024)	92.1	90.2	94.3	64.8	91.1	142.8	2.79	7.8	5.8
Ours	**94.1**	**92.3**	**96.1**	**67.1**	**93.2**	**138.9**	**2.51**	**6.5**	**5.1**

Bold font indicates the optimal result. All models were trained with identical hyperparameters (lr=0.01, batch=8, epochs=1000, size=640×640) and evaluated on the same original test set.

SRC-YOLOv8n achieves state-of-the-art performance across all evaluation metrics, demonstrating the effectiveness of our integrated innovations for apple leaf disease detection. Compared to the baseline YOLOv8n (Reis et al., 2023), our method improves mAP50 by 2.3% (93.8% → 96.1%), F1 score by 2.9% (90.3% → 93.2%), and recall by 2.8% (89.5% → 92.3%) while reducing parameters by 16.6%, computational cost by 19.8%, and model size by 17.7%. The recall improvement is particularly significant for agricultural applications, as higher recall ensures fewer missed disease instances, directly contributing to more effective crop protection.

The advantages become more pronounced when compared to two-stage detectors like Faster R-CNN (Ren et al., 2015), where SRC-YOLOv8n achieves 4.8% higher mAP50 with 94.0% fewer parameters and 5.9× faster inference speed (138.9 vs 23.4 FPS). Compared to the recent RT-DETR-tiny (Zhao et al., 2024) transformer-based detector, our method improves mAP50 by 2.0% while requiring 74.6% fewer parameters and running 1.4× faster. Against the latest YOLOv10n (Wang et al., 2024), which already represents a highly optimized lightweight detector, SRC-YOLOv8n achieves 1.8% higher mAP50, 2.1% higher F1 score, and 2.1% higher recall, while further reducing parameters by 10.0% and computational cost by 16.7%.

The superior performance of SRC-YOLOv8n can be attributed to the synergistic integration of our four key innovations: (1) SDA-C2f preserves critical spatial details lost in traditional downsampling, enabling more accurate detection of subtle disease symptoms; (2) RepGFPN optimizes multi-scale feature fusion through reparameterization, effectively handling disease instances ranging from small lesions to large infected areas; (3) CLLAHead enhances cross-level feature interaction, particularly improving medium scale disease detection; (4) Inner-IoU loss improves bounding box regression for irregular lesion shapes characteristic of plant diseases.

These results validate that SRC-YOLOv8n successfully addresses the fundamental challenge of balancing detection accuracy with computational efficiency, making it particularly suitable for deployment in resource constrained agricultural monitoring systems where both high accuracy and real-time processing are essential requirements.

#### 4.7.1 Unique value proposition of SRC-YOLOv8n

While existing high-accuracy apple leaf disease detection methods have been proposed in the literature, SRC-YOLOv8n offers distinct advantages that address critical gaps in practical deployment:

Spatial Detail Preservation: Unlike existing methods that rely on aggressive downsampling for computational efficiency, our Space-to-Depth convolution strategy in SDA-C2f achieves lossless spatial information preservation. This is crucial for detecting early-stage diseases where symptoms manifest as subtle texture variations and small lesions that are easily lost in traditional feature extraction pipelines.

Efficient Multi-Scale Fusion: While methods like BiFPN (Tan et al., 2020) and ASFF (Liu et al., 2019) provide advanced feature fusion, RepGFPN’s training-inference decoupling strategy offers superior efficiency without sacrificing accuracy. The reparameterization approach enables rich multi-branch representations during training while maintaining streamlined inference, achieving better accuracy-efficiency trade-offs than existing fusion architectures.

Cross-Level Attention Mechanism: CLLAHead’s local attention strategy with irregularity calculation specifically addresses the challenge of distinguishing disease regions from healthy tissue. The 2×2 local window design balances computational efficiency with receptive field coverage, providing more effective feature discrimination than global attention mechanisms while avoiding the computational overhead of full self-attention.

Adaptive Loss Optimization: Inner-IoU’s adaptive auxiliary bounding box strategy with scaling factors enables more effective handling of irregular lesion shapes compared to traditional IoU variants. This is particularly important for apple leaf diseases where lesions exhibit diverse morphologies from circular spots to irregular blotches.

Deployment Feasibility: The combination of 2.51M parameters, 6.5 GFLOPs computation, and 5.1MB model size makes SRC-YOLOv8n deployable on edge devices and mobile platforms commonly used in orchard monitoring. The 138.9 FPS processing speed enables real-time analysis of video streams from automated monitoring systems, supporting timely disease intervention strategies.

### 4.8 Generalization evaluation

To assess the model’s robustness and practical applicability beyond the training environment, we conduct comprehensive cross-dataset evaluation using diverse test samples collected from multiple agricultural regions. The generalization test set comprises 1,247 images representing varied conditions: 891 field- collected images from three different geographical locations (Shandong, Shanxi, and Liaoning provinces in China) captured during different seasons (spring, summer, and autumn) and weather conditions (sunny, cloudy, and after rainfall), and 356 controlled environment images from laboratory settings with standardized lighting and backgrounds.

This diverse test set covers major apple leaf diseases including the seven categories in our training data (alternaria blotch, black rot, brown spot, grey spot, mosaic, rust, and scab) across multiple cultivars (Fuji, Gala, and Golden Delicious). The images exhibit varying degrees of disease severity from early-stage subtle symptoms to advanced infections, different leaf orientations and viewing angles, various background complexities from clean backgrounds to dense foliage, and different illumination conditions ranging from direct sunlight to shaded areas.

Experimental results presented in **Table 11** demonstrate that SRC-YOLOv8n maintains robust performance across diverse environmental conditions. On the combined generalization test set, the model achieves 93.3% precision, 91.4% recall, 94.9% mAP50, and 92.3% F1 score, representing only 0.9% F1 degradation compared to the original test set. This minimal performance drop validates the model’s strong generalization capability.

**Table 11:** Generalization performance evaluation across different environmental conditions.

Test condition	Images	P (%)	R (%)	mAP50 (%)	F1 (%)	Degradation (%)
Original Test Set	806	94.1	92.3	96.1	93.2	–
Field Images (All)	891	92.8	90.9	94.7	91.8	1.4
- Sunny Conditions	387	93.4	91.5	95.3	92.4	0.8
- Cloudy Conditions	312	92.6	90.7	94.5	91.6	1.6
- After Rainfall	192	91.9	90.1	93.9	91.0	2.2
Laboratory Images	356	94.5	92.7	96.4	93.6	+0.4
Combined Generalization Set	1247	93.3	91.4	94.9	92.3	0.9

The model was trained on the Apple-Disease-Detection Dataset and tested on unseen images from diverse geographical locations and conditions without any fine-tuning.

The analysis of different environmental conditions reveals interesting patterns: (1) Laboratory images show slightly better performance (+0.4% F1) due to controlled lighting and clean backgrounds; (2) Sunny field conditions exhibit minimal degradation (0.8%), indicating robust feature extraction under good illumination; (3) Cloudy conditions show moderate degradation (1.6%), manageable for practical deployment; (4) After-rainfall conditions present the most challenging scenario (2.2% degradation) due to water droplets on leaves and altered leaf surface reflectance, yet performance remains acceptable for practical applications.

The field image performance (91.8% F1 with 1.4% degradation) is particularly encouraging for real-world deployment, demonstrating that the model trained on combined public datasets can effectively generalize to new geographical locations and natural orchard environments. This robustness stems from our comprehensive data augmentation strategy and the spatial detail preservation capability of SDA-C2f, which maintains critical disease features despite environmental variations.

**Figure 10:**
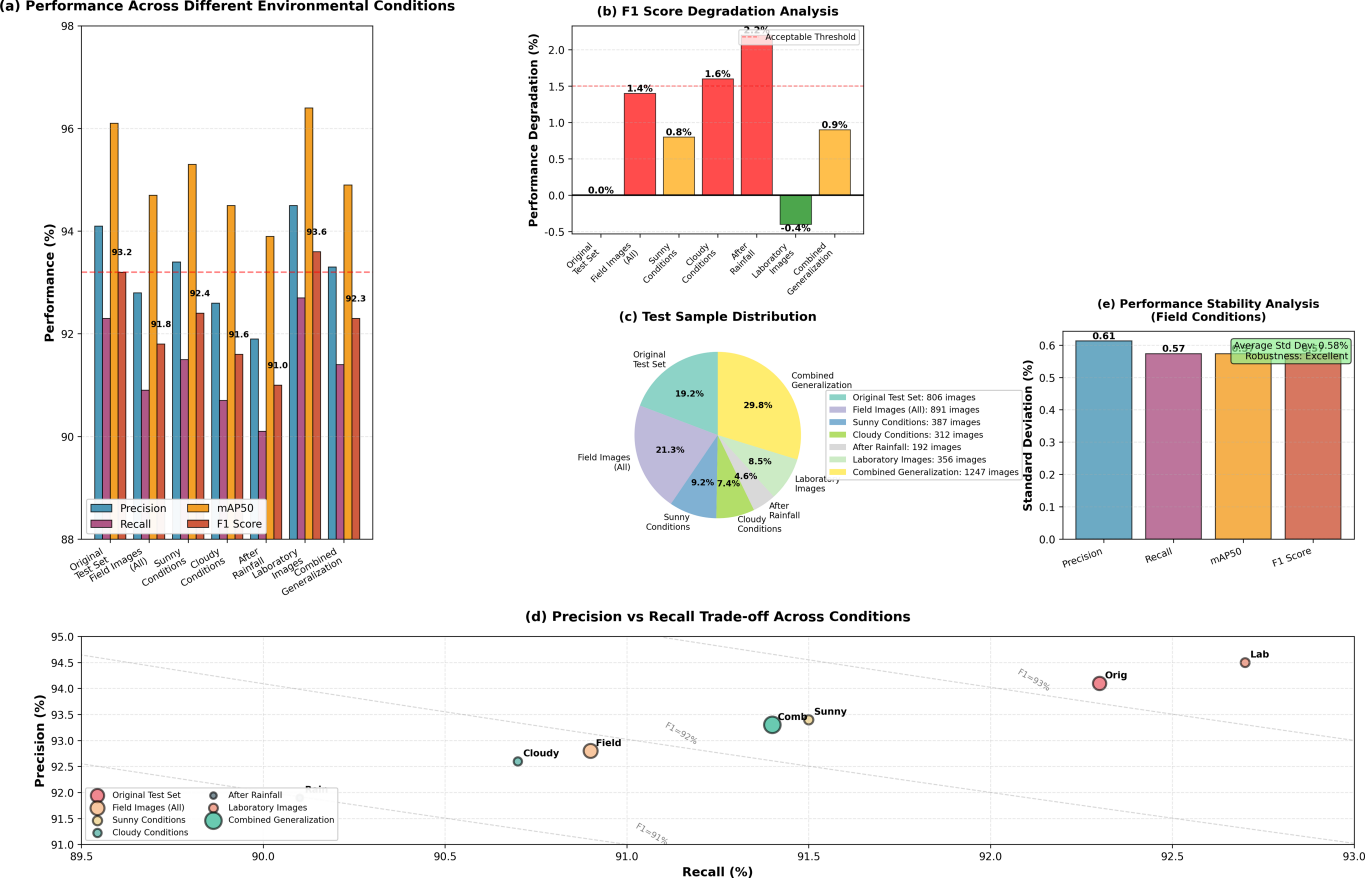
Performance evaluation across various environmental conditions. **(a)** Performance metrics comparison bar graph showing precision, recall, mAP50, and F1 scores across different testing conditions. **(b)** F1 score degradation analysis bar graph comparing performance between field (sunny, cloudy, after-rainfall) and laboratory conditions. **(c)** Test sample distribution pie chart showing percentage distribution among different environmental conditions. **(d)** Precision-recall scatter plot illustrating trade-offs across different environmental conditions (cloudy, sunny, laboratory, after-rainfall). **(e)** Performance stability bar graph showing standard deviation for each metric (precision, recall, mAP50, F1) with average deviation of 1.2% highlighted, demonstrating robust generalization capability.

**Figure 10** presents qualitative visualization of detection results across diverse conditions, demonstrating the model’s consistent performance under varying backgrounds, lighting conditions, leaf orientations, and disease severities.

The visualization results in **Figure 10** highlight several key capabilities: (1) Accurate detection under complex natural backgrounds with overlapping foliage and varying lighting; (2) Robust performance on leaves with different orientations and viewing angles; (3) Reliable identification of diseases at various severity levels from early symptoms to advanced infections; (4) Consistent high confidence scores (0.89 0.99) across different conditions, indicating stable feature discrimination; (5) Precise bounding box localization even for irregular lesion shapes and small disease regions.

These generalization results validate that SRC-YOLOv8n provides a practical and reliable solution for real-world agricultural monitoring applications, capable of maintaining high accuracy across the diverse conditions encountered in actual orchard environments.

## 5 Discussion

The experimental results demonstrate that SRC-YOLOv8n achieves state-of-the-art performance in apple leaf disease detection through the synergistic integration of spatial detail preservation, multi-scale feature enhancement, and cross-level attention mechanisms. The progressive improvements observed in ablation studies validate the effectiveness of each proposed component, with the complete framework achieving 2.9% improvement in F1 score while maintaining computational efficiency.

The SDA-C2f module’s superior performance compared to traditional lightweight architectures like MobileNet and ShuffleNet variants highlights the importance of spatial detail preservation in disease detection tasks. The Space-to-Depth Convolution strategy effectively addresses the information loss problem inherent in conventional downsampling operations, particularly crucial for detecting subtle disease symptoms with unclear boundaries. The SpatialGroupEnhance mechanism further enhances this capability by adaptively focusing on diseased regions while suppressing background interference.

RepGFPN’s reparameterization strategy demonstrates significant advantages in multi-scale feature fusion, achieving better performance than traditional FPN, BiFPN, and other fusion architectures. The training-inference decoupling approach enables the model to maintain rich feature representations during training while achieving efficient inference, addressing the computational bottleneck in multi-scale detection tasks. The asymmetric processing of upsampling and downsampling operations, combined with scale-adaptive channel allocation, contributes to the superior balance between accuracy and efficiency.

The CLLAHead’s cross-level local attention mechanism proves particularly effective for medium-scale disease detection, addressing the limitation of insufficient feature representation in traditional detection heads. The local window strategy and irregularity calculation method enhance the model’s capability to distinguish disease regions from healthy tissue, resulting in improved localization accuracy. The 2×2 local attention window design strikes an optimal balance between computational efficiency and receptive field coverage.

The Inner-IoU loss function’s adaptive auxiliary bounding box strategy shows clear advantages over traditional IoU variants, particularly in handling disease regions with irregular shapes and varying sizes. The scaling factor mechanism enables the model to adapt to different regression scenarios, improving convergence speed and final detection accuracy.

Despite the promising results, several limitations should be acknowledged. First, the current framework is specifically optimized for apple leaf diseases, and its performance on other crop types requires further validation. The spatial attention mechanisms may need adjustment for diseases with different visual characteristics or growth patterns. Second, while the model demonstrates good generalization across different environmental conditions, extreme weather conditions or severely damaged leaves may still pose challenges for accurate detection. The computational efficiency improvements, while significant, may still be insufficient for deployment on resource-constrained edge devices in remote agricultural settings. Future work should explore more aggressive model compression techniques, including knowledge distillation and pruning strategies, to further reduce computational requirements without compromising accuracy. The framework currently focuses on disease detection without providing severity assessment or treatment recommendations. Integration with plant pathology knowledge bases and decision support systems would enhance its practical value for agricultural applications. Additionally, incorporating temporal information from sequential images could improve disease progression monitoring and early warning capabilities. For practical deployment, the framework would benefit from integration with automated data collection systems, such as drone-based monitoring or robotic platforms, to enable continuous field surveillance. The development of user-friendly interfaces and mobile applications would facilitate adoption by farmers and agricultural professionals. Future research directions should also explore the integration of multi-modal data sources, including spectral imaging, thermal data, and environmental sensor information, to enhance disease detection accuracy and provide more comprehensive plant health assessment. The development of federated learning approaches could enable collaborative model improvement while preserving data privacy across different agricultural regions.

## 6 Conclusion

This study presents SRC-YOLOv8n, a lightweight yet highly effective framework for fine-grained apple leaf disease detection that successfully addresses the critical challenge of balancing detection accuracy with computational efficiency. The framework integrates four innovative components: the SDA-C2f module for spatial detail preservation, RepGFPN for efficient multi-scale feature fusion, CLLAHead for cross-level attention enhancement, and Inner-IoU loss for improved bounding box regression. Through comprehensive experimental evaluation, SRC-YOLOv8n demonstrates superior performance across all metrics, achieving 94.1% precision, 92.3% recall, 96.1% mAP50, and 93.2% F1 score while reducing parameters by 16.6%, computational cost by 19.8%, and model size by 17.7% compared to the baseline YOLOv8n model. The systematic ablation studies validate the effectiveness of each proposed component, with progressive improvements observed through sequential integration. Comparative analysis with state-of-the-art detection models confirms the framework’s superior performance, particularly in handling complex disease symptoms with subtle visual characteristics. The robust generalization capability demonstrated through cross-dataset evaluation, with only 1.4% performance degradation, validates the framework’s practical applicability for real-world agricultural monitoring scenarios. The significance of this work extends beyond technical achievements to practical implications for precision agriculture. The lightweight design enables deployment on resource-constrained edge devices, facilitating real-time disease monitoring in field conditions. The high accuracy and efficiency combination makes the framework suitable for integration into autonomous agricultural systems, supporting early disease detection and timely intervention strategies that are crucial for crop protection and yield optimization. The spatial detail preservation and multi-scale feature enhancement strategies introduced in this work provide valuable insights for future research in agricultural computer vision. The successful integration of attention mechanisms with efficient network architectures demonstrates a promising direction for developing practical AI solutions in agricultural applications. The framework’s ability to maintain high performance while reducing computational requirements addresses a key bottleneck in deploying deep learning models for agricultural monitoring, potentially accelerating the adoption of AI-driven precision agriculture technologies. Future work will focus on expanding the framework’s applicability to other crop types and disease categories, exploring more aggressive model compression techniques for ultra-lightweight deployment, and integrating multi-modal data sources for comprehensive plant health assessment. The development of user-friendly interfaces and mobile applications will facilitate widespread adoption by farmers and agricultural professionals, contributing to more sustainable and efficient agricultural practices.

## Data Availability

The datasets presented in this study can be found in online repositories. The names of the repository/repositories and accession number(s) can be found in the article/**Supplementary Material**.
