# Supplementary figures and images for: SRC-YOLOv8n: a lightweight framework for fine-grained apple leaf disease detection with spatial detail preservation and multi-scale feature enhancement
Source: Front Plant Sci. 2026 Feb 16;16:1709939. doi: 10.3389/fpls.2025.1709939 (PMC12950693; doi:10.3389/fpls.2025.1709939)

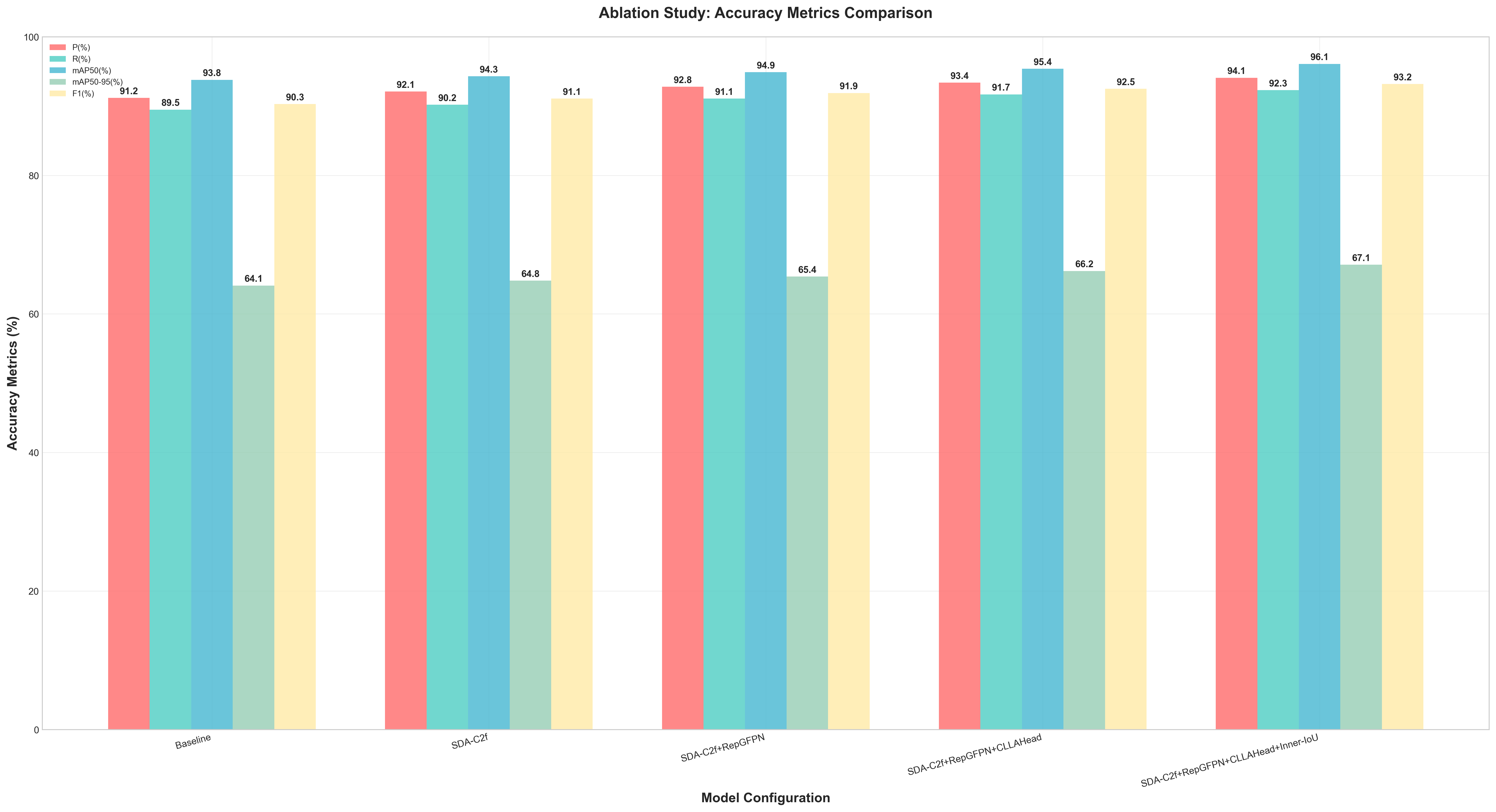

Supplement: Supplementary file 1 [file Image1.png]

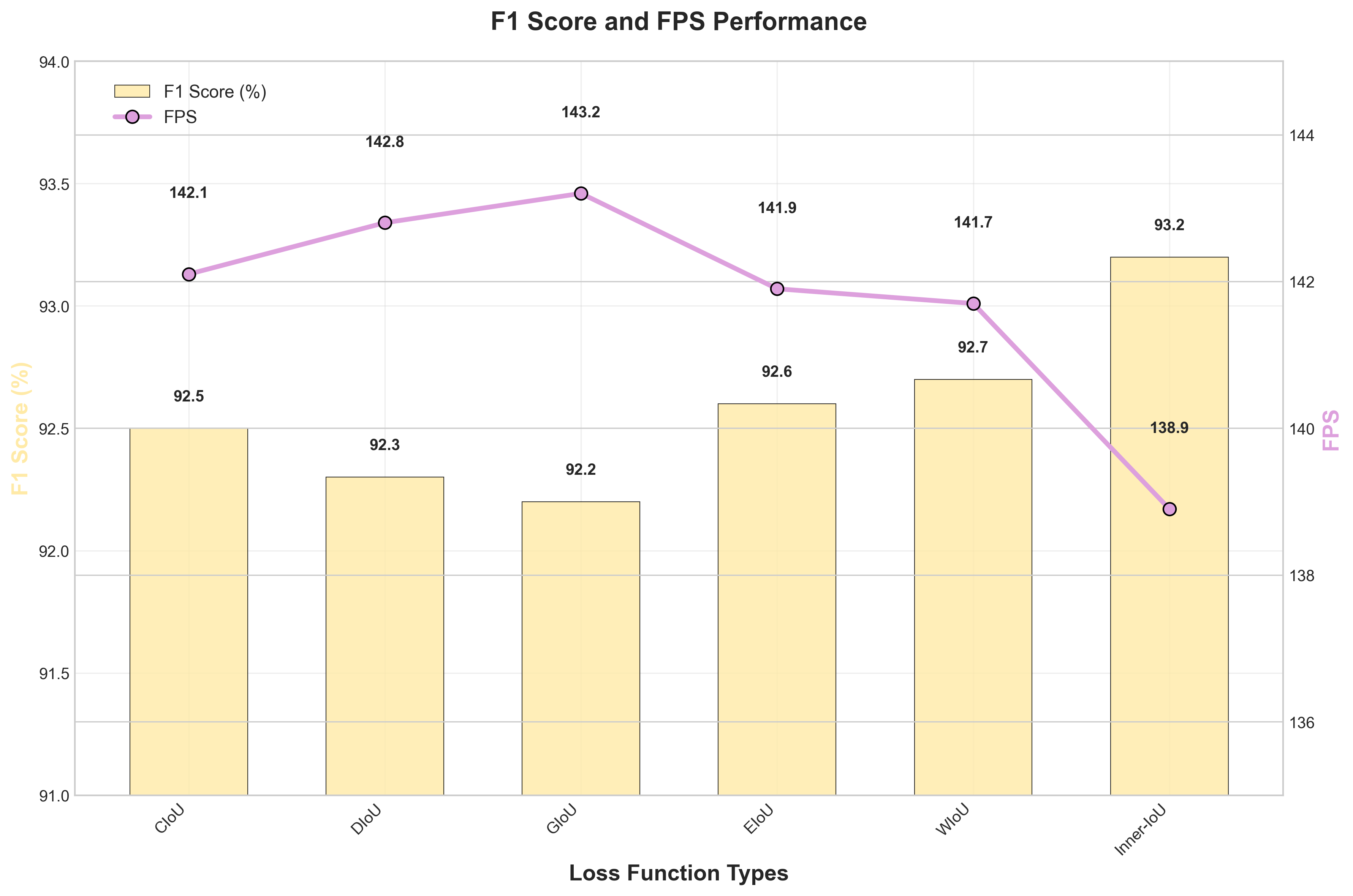

Supplement: Supplementary file 2 [file Image2.png]

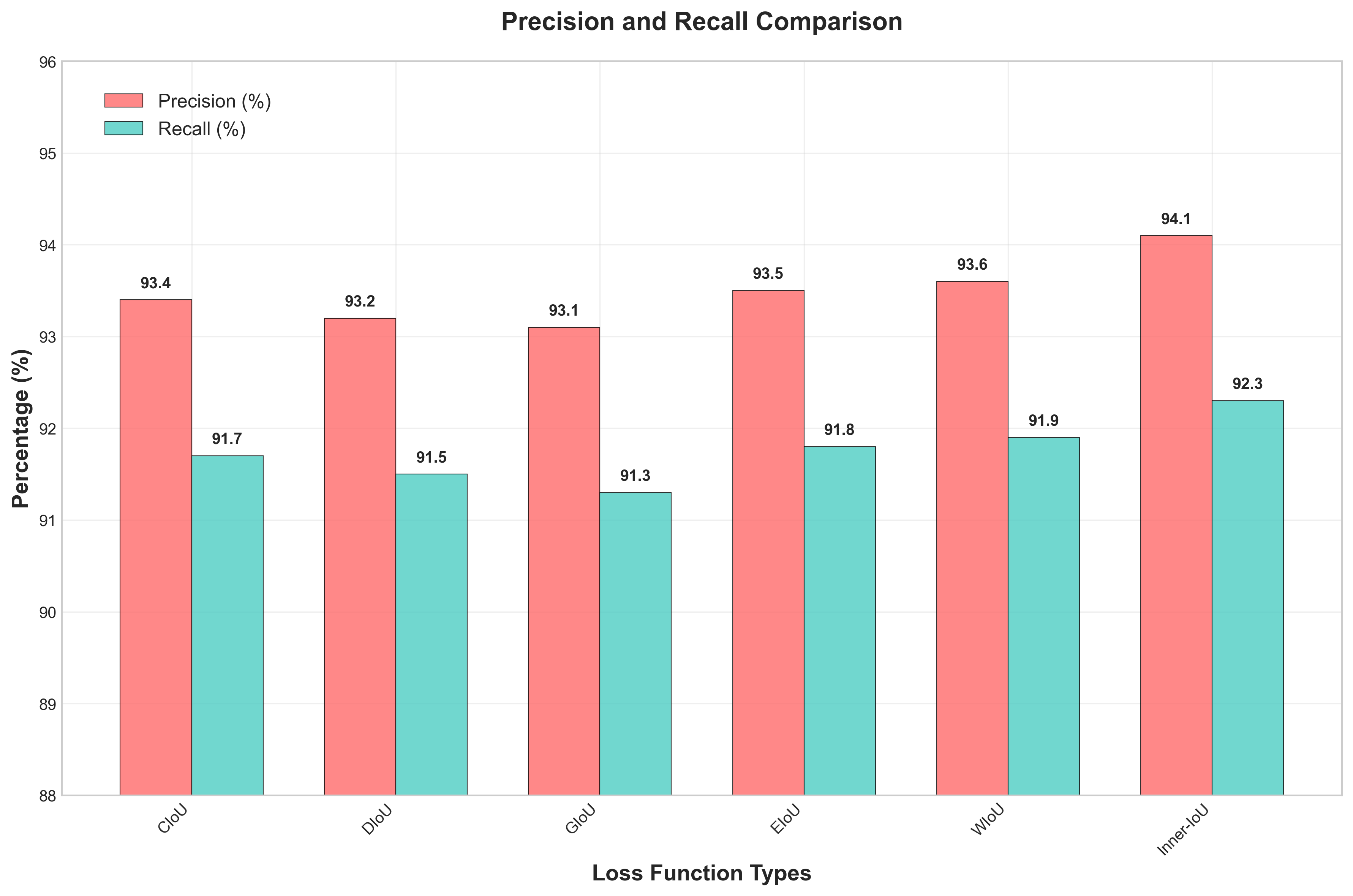

Supplement: Supplementary file 3 [file Image3.png]

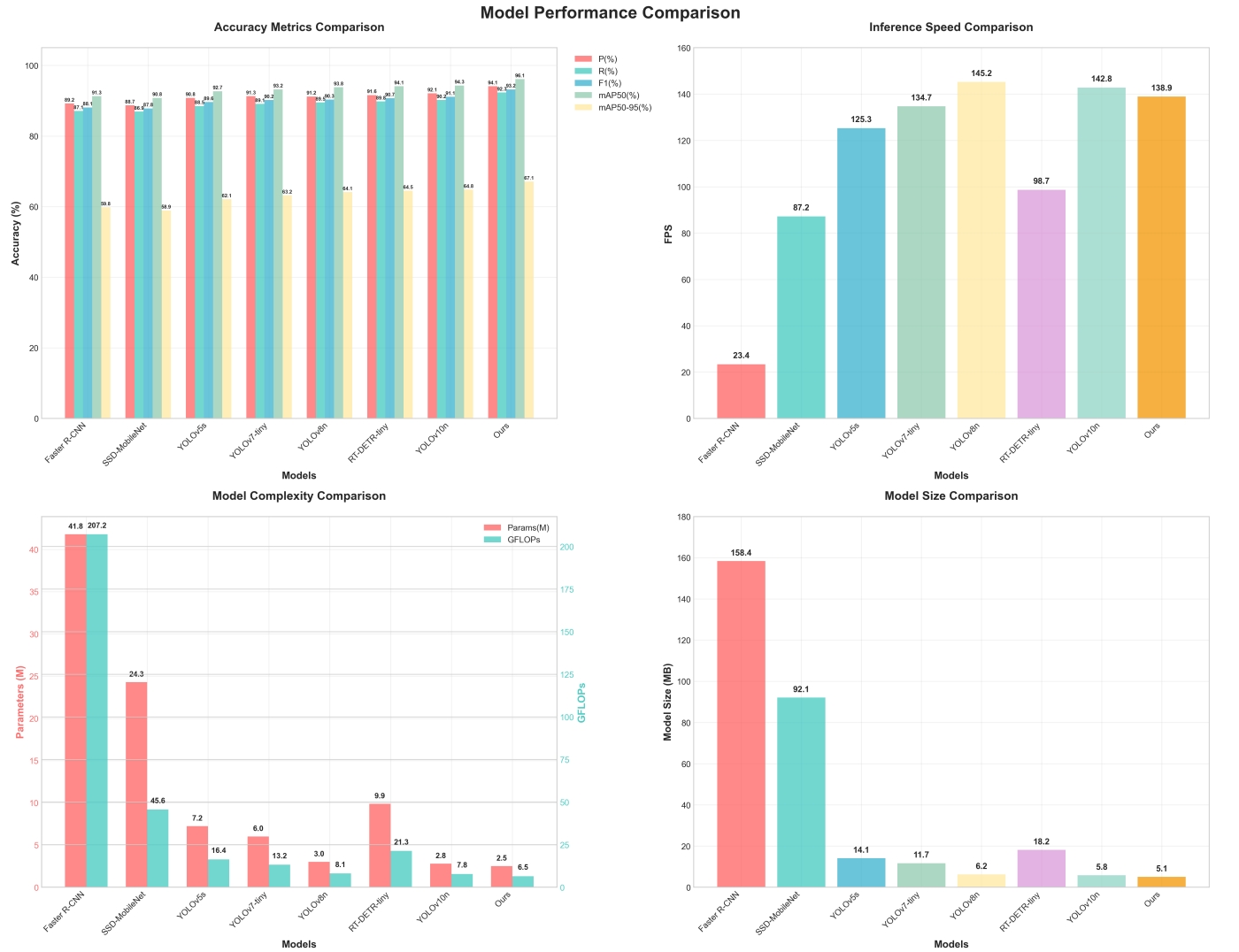

Supplement: Supplementary file 4 [file Image4.png]

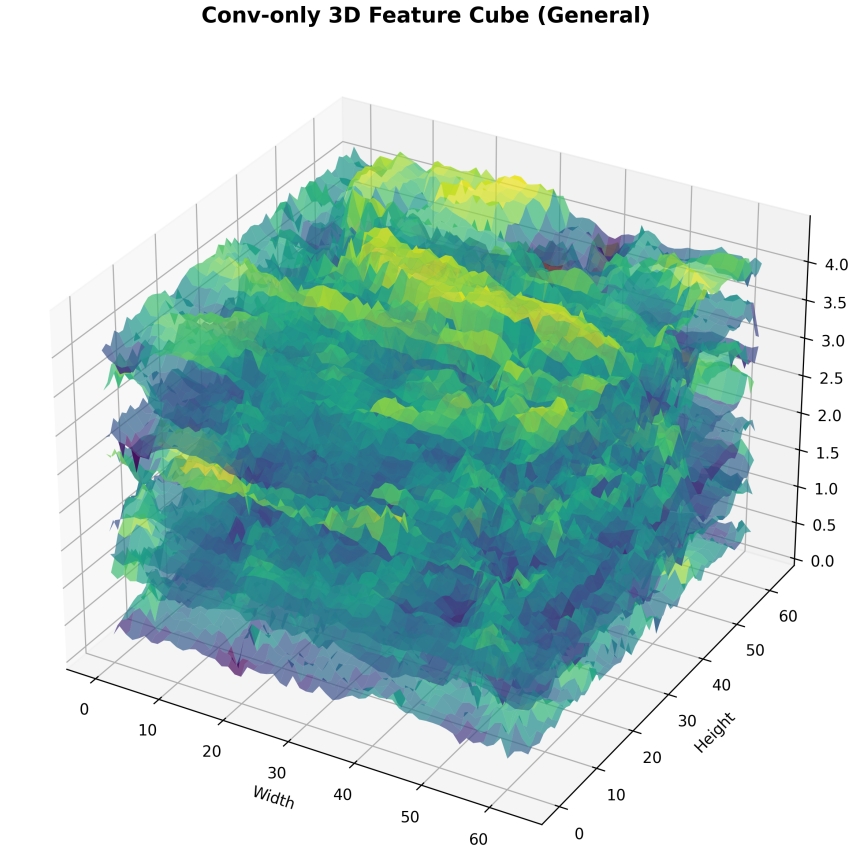

Supplement: Supplementary file 5 [file Image5.png]

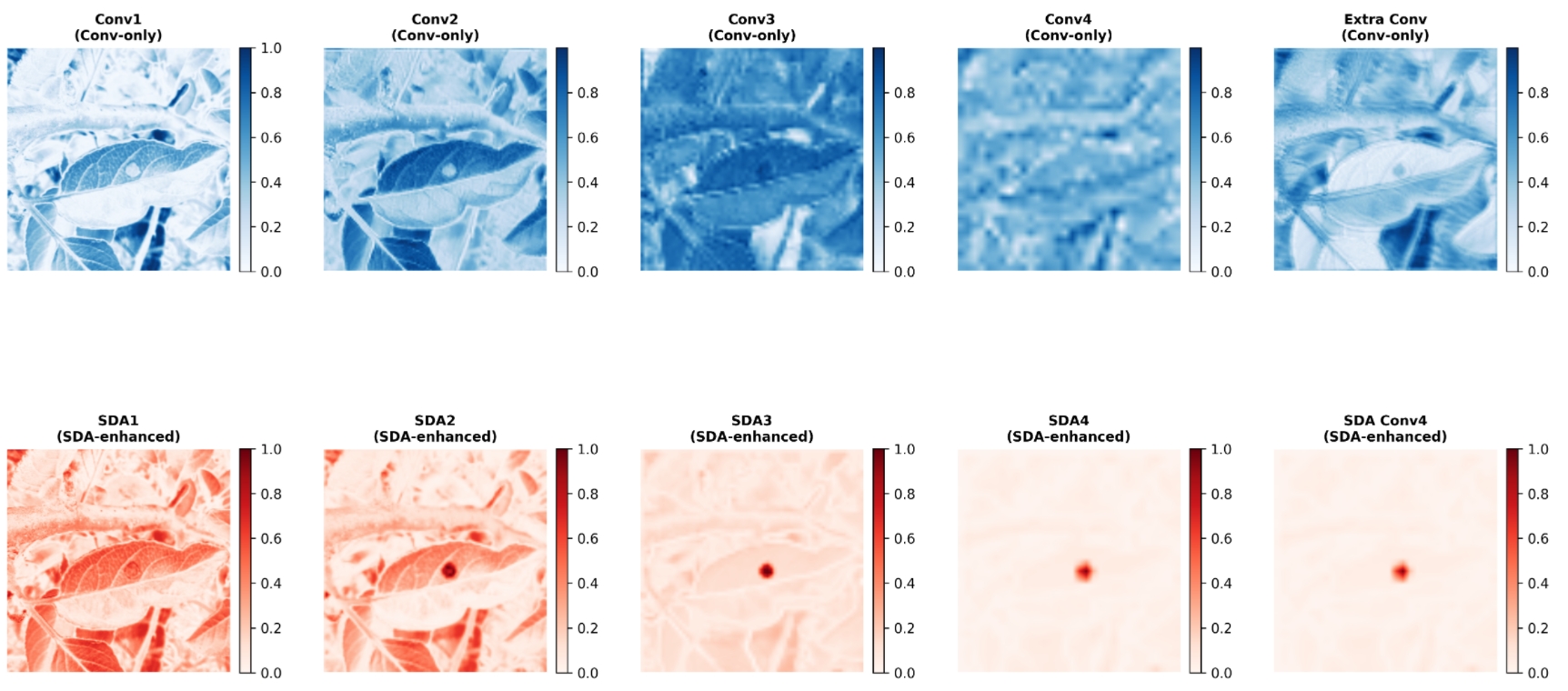

Supplement: Supplementary file 6 [file Image6.png]

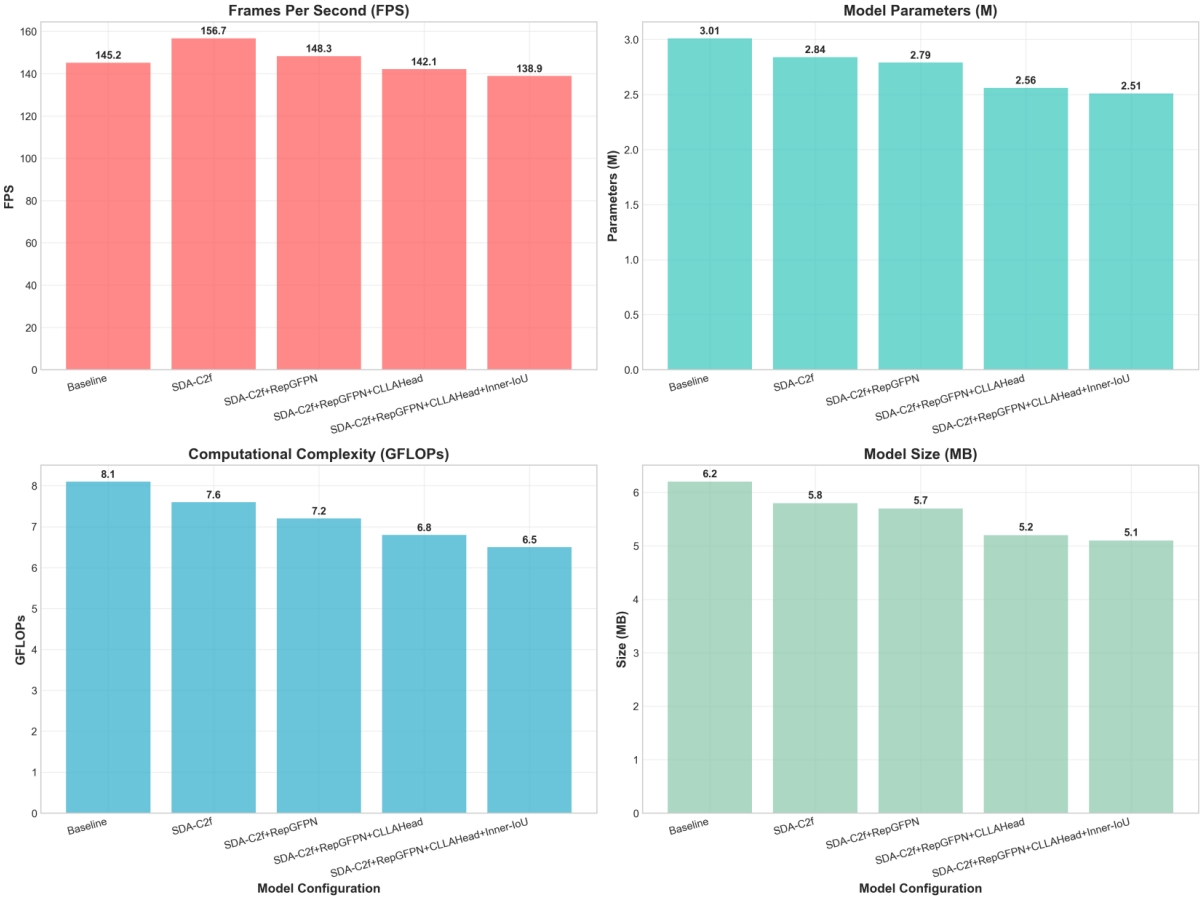

Supplement: Supplementary file 7 [file Image7.png]

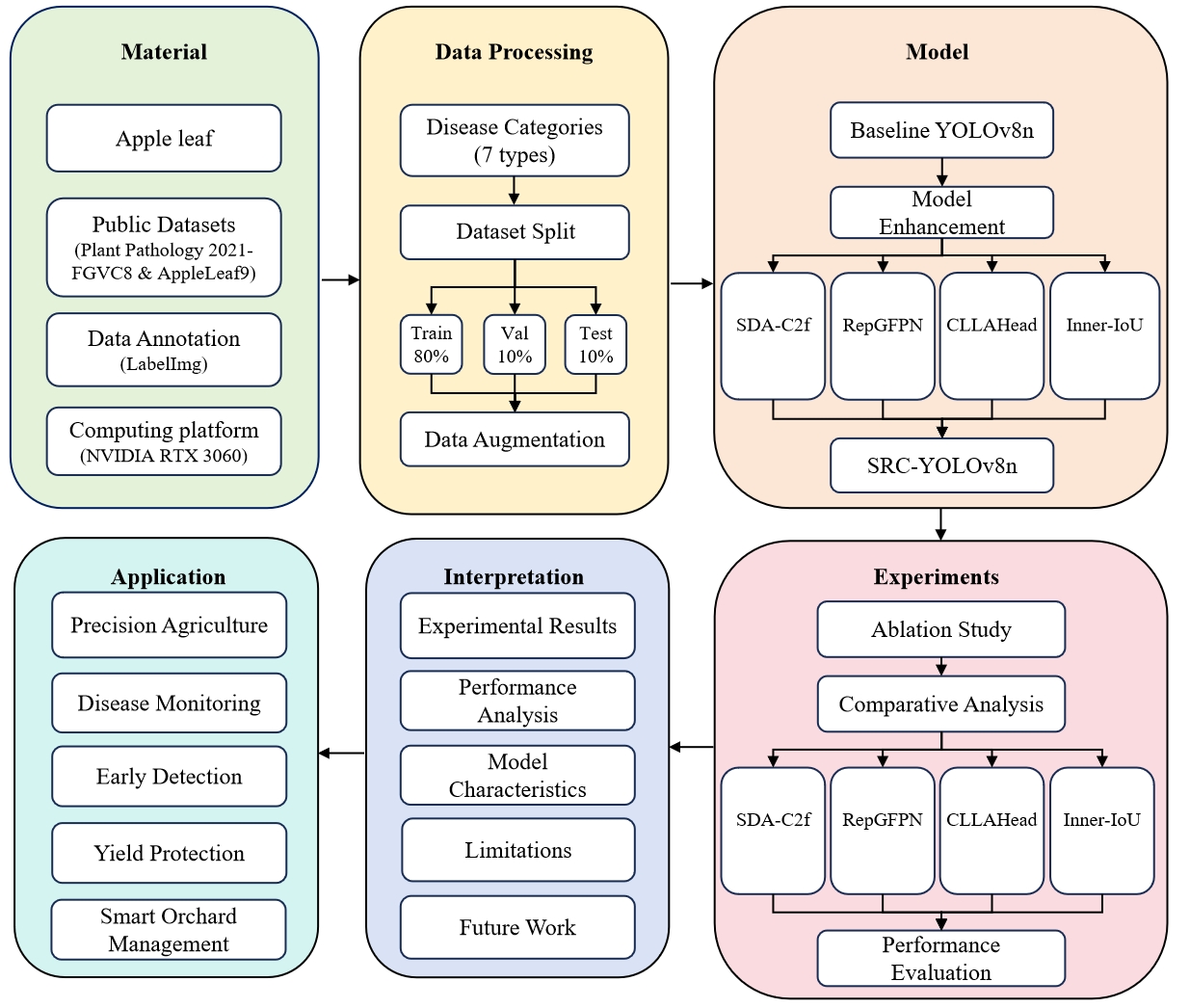

Supplement: Supplementary file 8 [file Image8.png]

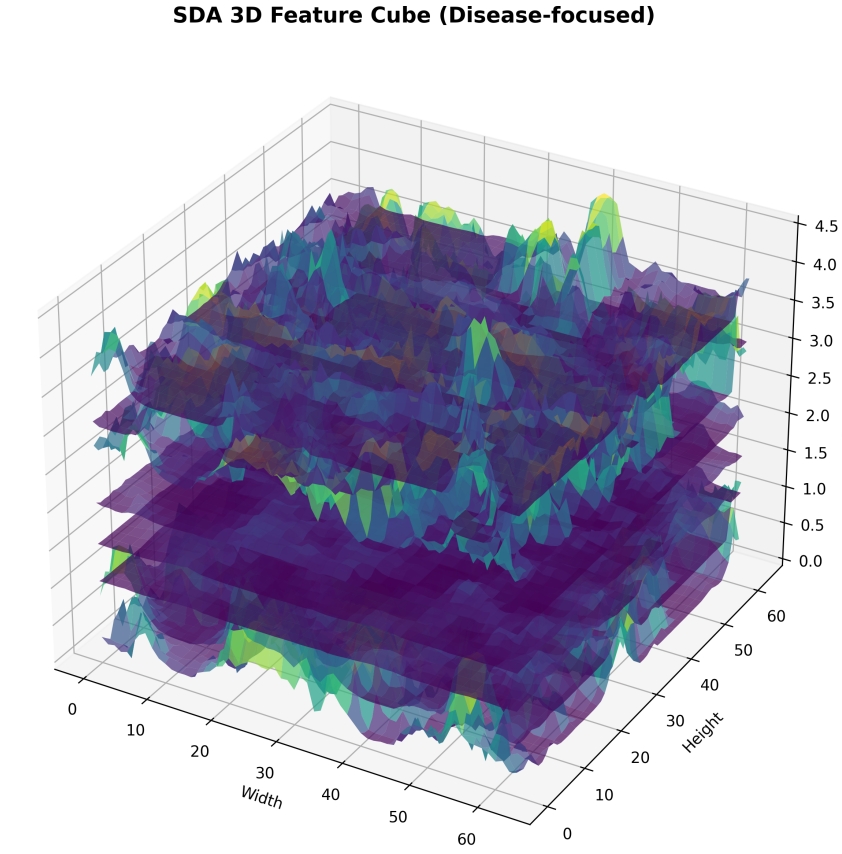

Supplement: Supplementary file 9 [file Image9.png]

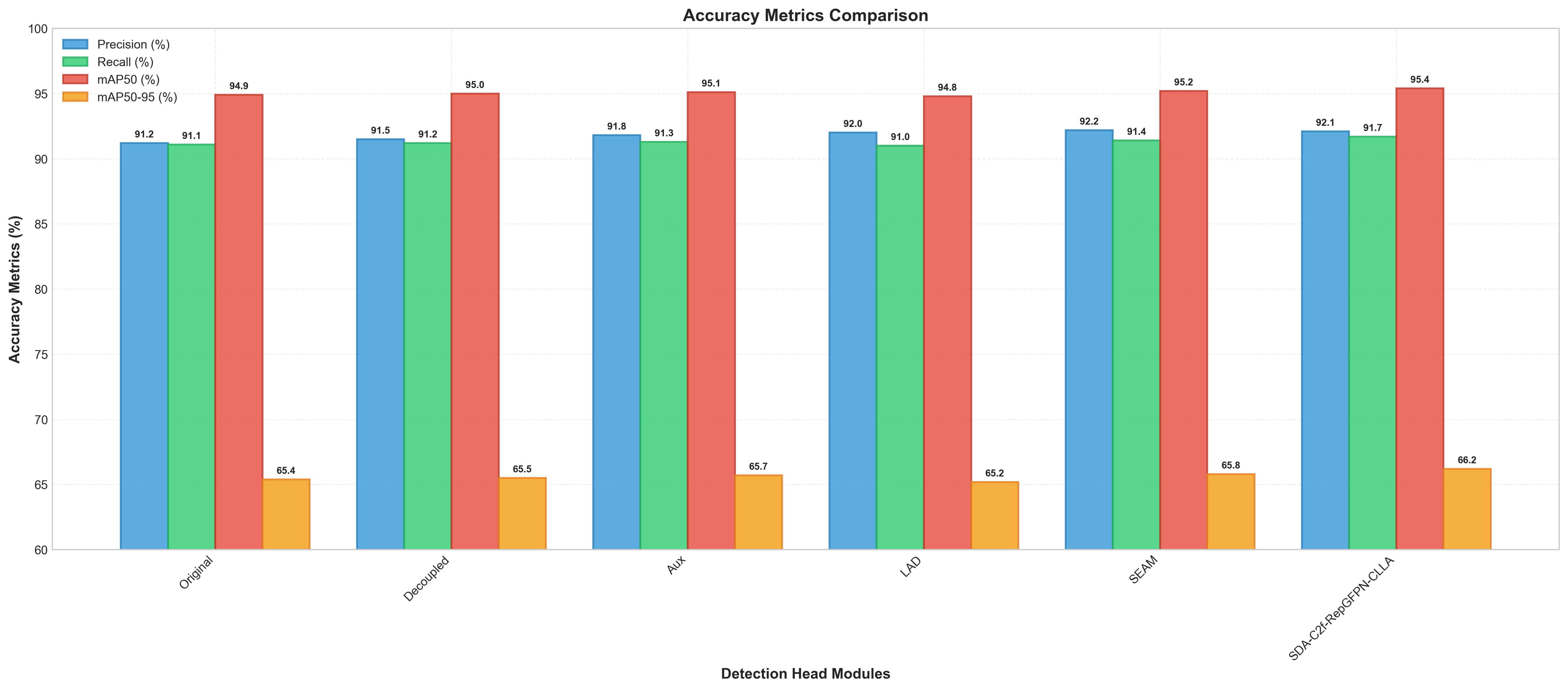

Supplement: Supplementary file 10 [file Image10.png]

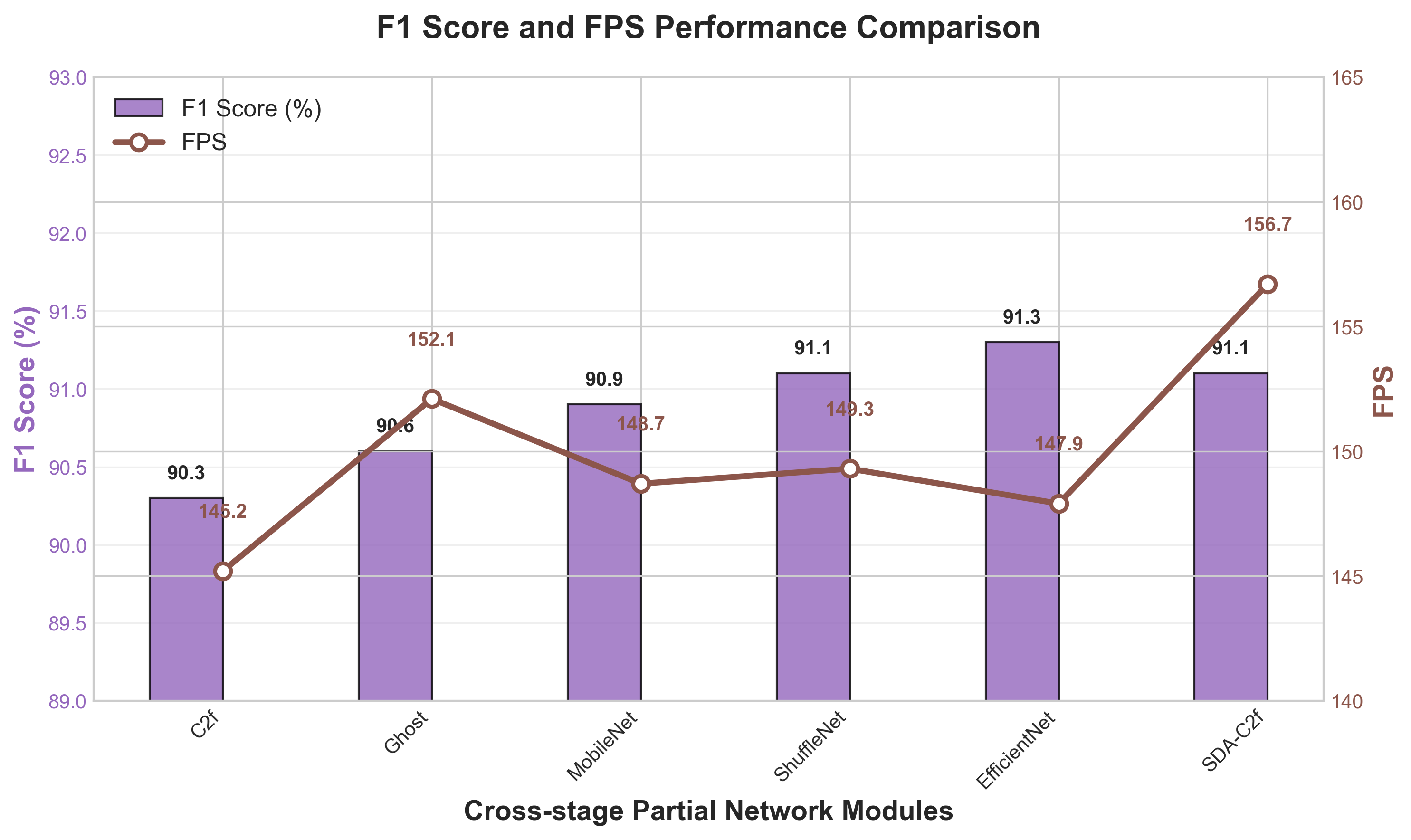

Supplement: Supplementary file 11 [file Image11.png]

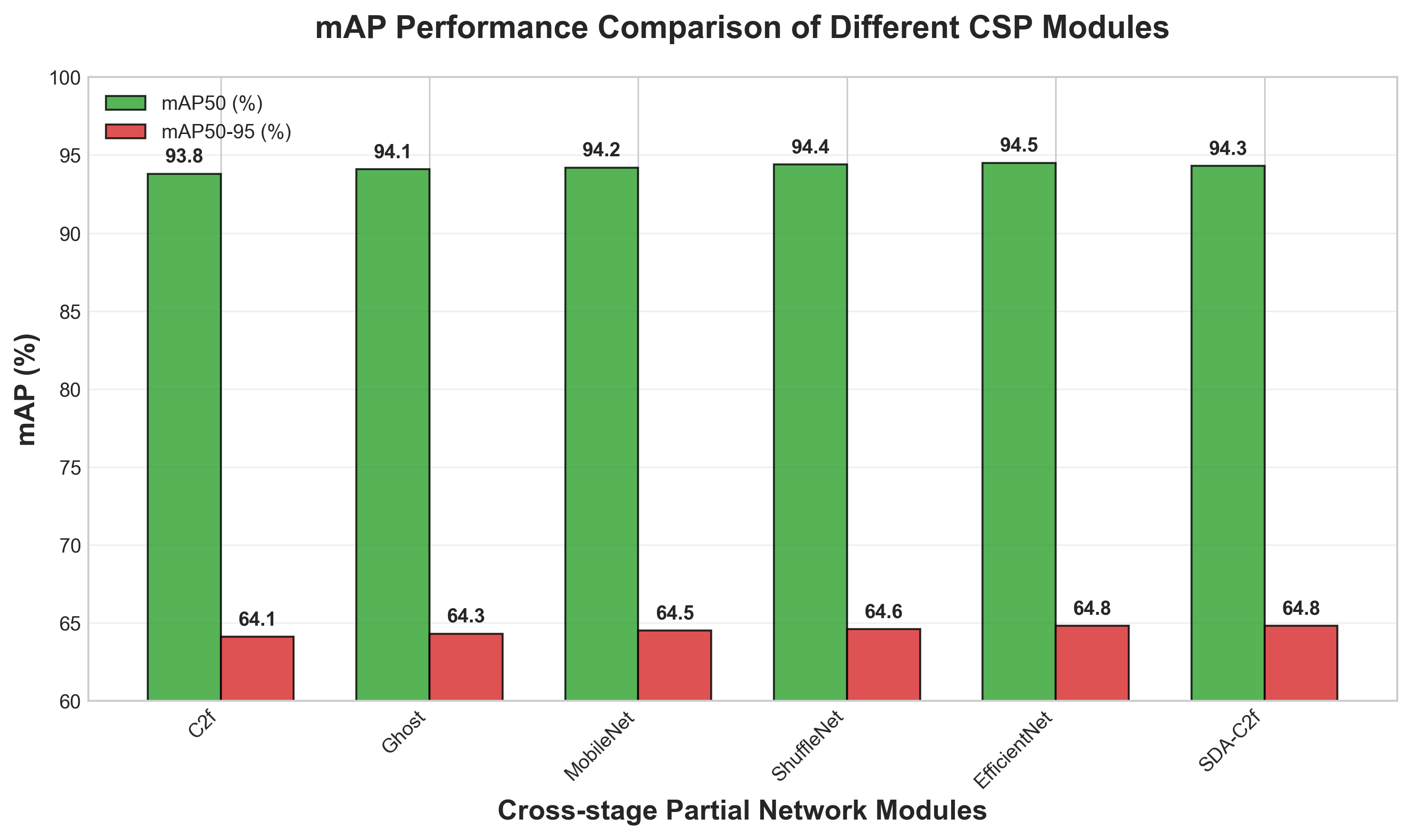

Supplement: Supplementary file 12 [file Image12.png]

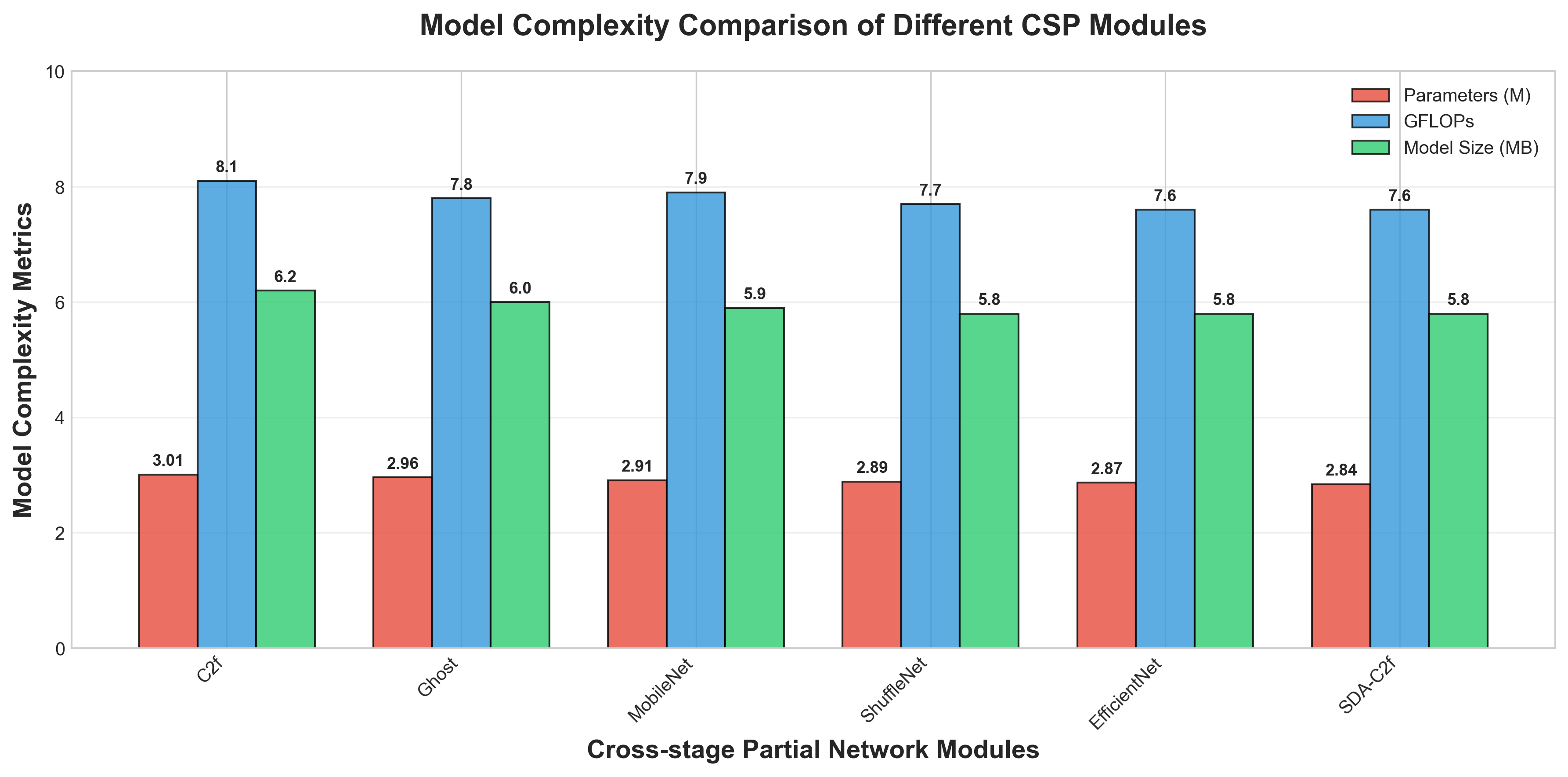

Supplement: Supplementary file 13 [file Image13.png]

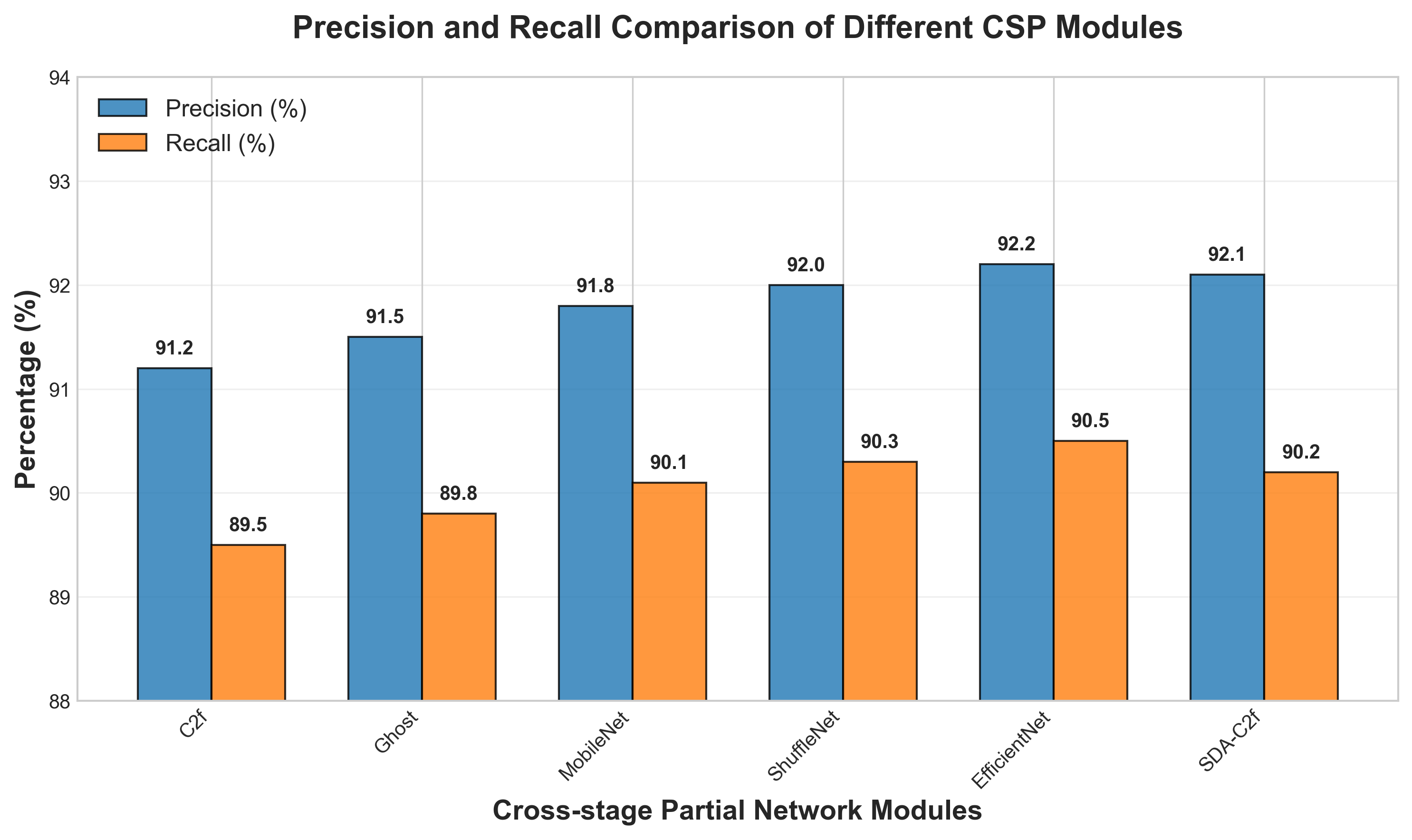

Supplement: Supplementary file 14 [file Image14.png]
